# Carotenoid‐Rich Pigment Extract From *Micrococcus terreus* SK34 Induces Apoptosis in SH‐SY5Y Neuroblastoma Cells: Integrated Metabolomic, Molecular Docking, and Experimental Validation

**DOI:** 10.1002/mbo3.70382

**Published:** 2026-09-25

**Authors:** Sinem Ilayda Karaagac, Seyda Albayrak, Azizeh ShadiDizaji, Muhammet Enes Kiziler, Elvan Eroglu, Nazli Pinar Arslan, Mesut Taskin

**Affiliations:** ^1^ Department of Molecular Biology and Genetics, Faculty of Science Ataturk University Erzurum Türkiye; ^2^ Department of Plant Biotechnology, Faculty of Agriculture Ataturk University Erzurum Türkiye; ^3^ Vaccine Development Application and Research Center Ataturk University Erzurum Türkiye; ^4^ Vocational School of Health Services Bingol University Bingol Türkiye; ^5^ Department of Basic Sciences, Faculty of Dentistry Ataturk University Erzurum Türkiye

**Keywords:** antibacterial, anticancer, apoptosis, carotenoids, *Micrococcus terreus* SK34, molecular docking

## Abstract

Bacteria‐derived carotenoids have attracted increasing attention as natural bioactive compounds with potential pharmaceutical applications, particularly in cancer therapy. However, the chemical composition and anticancer mechanisms of carotenoids from *Micrococcus terreus* remain largely unexplored. This study aimed to characterize the carotenoid extract from the locally isolated *M. terreus* SK34 strain (GenBank: PX765938) and to investigate its anticancer activity and underlying mechanisms using integrated in vitro and in silico approaches. Under nonoptimized culture conditions, strain SK34 produced 128 mg/L of carotenoid‐rich pigment extract. LC‐Q‐TOF‐MS analysis identified eight carotenoids, including phytoene, β‐carotene, astaxanthin, zeaxanthin, β‐cryptoxanthin, α‐carotene, lycopene, and sarcinaxanthin. The extract exerted selective cytotoxicity against different cancer cell lines (IC_50_ = 61.91–243.76 μg/mL), particularly the neuroblastoma (SH‐SY5Y) cancer cell line, while showing minimal cytotoxicity toward normal human dermal fibroblasts. Gene expression analysis demonstrated the significant upregulation of proapoptotic genes (p53, BAX, CASP3, CASP8, and CASP9) in the SH‐SY5Y cell line, together with the downregulation of antiapoptotic genes (BCL‐2 and AKT1), whereas autophagy‐related genes were not significantly affected. ELISA and flow cytometry analyses further confirmed apoptosis in the SH‐SY5Y cell line. Molecular docking analyses revealed that β‐carotene, astaxanthin, and zeaxanthin exhibited strong binding affinities toward apoptosis‐related proteins, supporting the experimental findings. This study provides the first comprehensive characterization of carotenoids from *M. terreus* SK34 and highlights their potential as selective apoptosis‐inducing agents for future anticancer therapy.

## Graphical abstract

Carotenoid extract from *Micrococcus terreus* SK34 was characterized by LC‐Q‐TOF‐MS and evaluated for its in vitro anticancer potential. The extract exhibited selective cytotoxicity against different cancer cell lines, particularly neuroblastoma cells (SH‐SY5Y), while sparing normal cells. Mechanistic analyses confirmed the induction of apoptosis, and molecular docking supported interactions between major carotenoids and apoptosis‐related proteins, highlighting their potential as promising candidates for anticancer therapies.

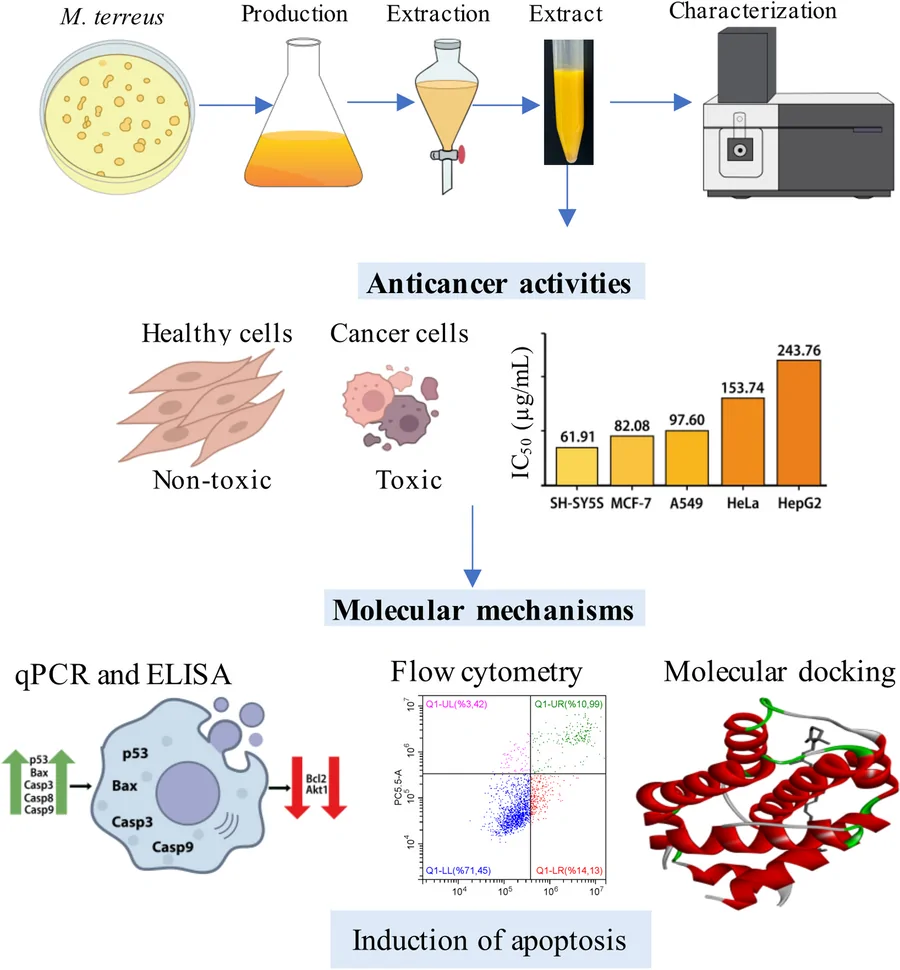

## 1 Introduction

Carotenoids are a group of isoprenoid pigments that can be synthesized by various organisms, such as microorganisms, macroalgae, and plants (Patel et al. 2022; Esim et al. 2024). Their general structural units consist of 6, 8, 9, and 10 C5 isoprenoid units, containing a linear hydrocarbon backbone, which are classified as C30, C40, C45, and C50, respectively. C40 is the most frequently found carotenoid in nature (Armstrong and Hearst 1996). Carotenoids comprise a diverse group of lipid‐soluble natural pigments. They exhibit different colorations, such as yellow, orange, and red. According to their molecular composition, these compounds are categorized into two main classes: Carotenes and Xanthophylls. The carotenes consist solely of carbon and hydrogen, while xanthophylls contain oxygen atoms as well as carbon and hydrogen atoms (Mussagy et al. 2019; Esim et al. 2024).

While approximately 50 different types of carotenoids are known to exist in nature, the most common are lycopene, torulens, and α‐, β‐, and γ‐carotenes. Examples of the main forms of carotenoids include zeaxanthin, astaxanthin, lutein, β‐cryptoxanthin, fucoxanthin, and peridinin (Taskin and Erdal 2011; Taskin et al. 2011; Esim et al. 2024; Arslan, Dawar, et al. 2025; Arslan, Azad, et al. 2025). Carotenoid producers include filamentous microfungi, micromontars, yeasts, and bacteria. Filamentous microfungi are reported in the literature to produce commonly found carotenoids, such as β‐carotene, torulene, lycopene, astaxanthin, and neurosporaxanthin (Avalos and Carmen Limón 2015).

Carotenoids are essential for phototropism, photon protection, and photosynthesis processes in microorganisms. These compounds also protect microorganisms against oxidative stress and increase the virulence of pathogenic microorganisms. Importantly, these naturally occurring compounds act as metabolic precursors in the synthesis of other metabolites in microorganisms (Saini and Keum 2019; Grivard et al. 2022; Kiziler et al. 2022; Saubenova et al. 2024). For example, carotenoids synthesized by photosynthetic and nonphotosynthetic bacteria exhibit photoprotective and antioxidant activities, protecting these bacteria from damage caused by ultraviolet (UV) radiation and sunlight. They also play a role in the adaptation response to low temperatures by regulating the fluidity of the bacterial membrane. Furthermore, they can protect pathogenic bacteria against antimicrobial agents by regulating the lipid composition of membranes (Saubenova et al. 2024).

In addition to their roles in physiological functions in bacteria, bacterially derived carotenoids have also been reported to exert beneficial effects in humans. For example, bacterial‐derived carotenoids can exhibit anticancer, photoprotective, UV protective, antimicrobial, antioxidant, antifungal, antibiofilm, antihemolytic, antiviral, antiobesity, antidiabetic, anti‐inflammatory, antilipidemic, wound‐healing, cardioprotective, antiaging, and provitamin A activities (Mohana et al. 2013; Mendes‐Silva et al. 2021; López et al. 2023; Al‐Monofy et al. 2025; Paredes Contreras et al. 2025; Taghipour et al. 2025; Karimian Dorhoni et al. 2026).

Examples of bacterial genera that synthesize carotenoids include *Agrobacterium, Arthrobacter, Bacillus, Corynebacterium, Flavobacterium, Kocuria palustris, Paracoccus, Rhodococcus, Halomonas*, and *Micrococcus*, all of which have been reviewed in the literature (Arslan, Azad, et al. 2025). The genus *Micrococcus* includes Gram‐positive and rod‐shaped species. While some strains cause infections, others are of biotechnological importance (Rostami et al. 2016; Shahin et al. 2022; Hegazy et al. 2024). For instance, *Micrococcus luteus* is one of the most studied and biotechnologically important species in this genus. It is stated that this species produces pigments, such as β‐carotene, sarquinaxanthin, and zeaxanthin, and these pigments exhibit different bioactive properties, such as antioxidant, anticancer, and antimicrobial properties (Shahin et al. 2022; Kandasamy and Kathirvel 2024; Hegazy et al. 2024). One of the least studied species in the *Micrococcus* genus is *Micrococcus terreus*. This species was first described as a new species in 2010 (Zhang et al. 2010). Another study indicated that the pigment extract obtained from this species exhibited anticancer properties on HeLa (the cervical cancer cell line), HepG2 (liver cancer cell line), and Jurkat (leukemia) (Shukla and Nadumane 2021). However, the anticancer efficacy of its pigments on other cancer types has not been tested. This is the first gap in the literature on *M. terreus‐*derived pigments. The second gap is that the pigment composition of this bacterium has not been established in the literature. The third gap is that the in silico analysis of the anticancer activities of its pigments has not yet been carried out. Herein, this study aims to characterize the pigment composition of locally isolated *M. terreus* and to evaluate the anticancer effects of its pigment extract on different cancer cell lines, as well as to investigate the underlying mechanisms of the pigment extract's anticancer actions through in silico analyses.

## 2 Materials and Methods

### 2.1 Isolation of Pigment‐Producing Bacteria From Soil Environments

Soil samples collected under sterile conditions from various districts of Erzurum province were brought to the laboratory as soon as possible and used in isolation studies. For the isolation of bacteria, 1 g of each soil sample was suspended in 9 mL of sterile physiological saline (0.85% NaCI) and homogenized thoroughly. Serial 10‐fold dilutions were subsequently prepared using sterile physiological saline (10^−1^–10^−9^). Subsequently, 0.1 mL of each dilution was spread onto Petri dishes containing tryptic soy agar (TSA) medium. The inoculated Petri dishes were incubated at different temperatures (20°C, 30°C, and 40°C) for 24–48 h under aerobic conditions. After incubation, colonies displaying distinct pigmentation and morphological characteristics were carefully selected, purified by repeated streaking on fresh TSA plates, and preserved for subsequent experiments (Yildirim et al. 2026).

### 2.2 Comparison of Pigment Production Abilities of Isolates

The precultures of bacterial isolates were prepared in 250 mL Erlenmeyer flasks containing 100 mL of tryptic soy broth (TSB) medium (at 30°C and 150 rpm for 24 h). The concentration of the preculture was then adjusted to yield an absorbance of 2.0 at 600 nm for each bacterial isolate. Production studies were also carried out in 250 mL Erlenmeyer flasks containing 100 mL of TSB medium. For this, 1 mL of the precultures was inoculated into the TSB in the Erlenmeyer flasks, and the flasks were incubated at 30°C and 150 rpm for 120 h. At the end of this period, the cultures were centrifuged, and pigment extraction was performed from the cell biomass.

Carotenoid extraction was performed by HCl pretreatment and acetone–methanol treatment. In brief, the centrifuged wet cell pellet was washed 2–3 times with pure water, and then 1 M HCl (in a 1:2 ratio) was added to the cells. The cell‐HCl suspension was vortexed for 5–10 min and then kept in a water bath at 50°C for 30 min to lyse the cells. The suspension was centrifuged, and the water phase (supernatant) was removed. A 90% acetone–methanol mixture in a 1:10 ratio was added to the wet cell pellet, the suspension was vortexed, and then centrifuged to collect the supernatant (colored phase) in a new falcon. Acetone–methanol treatment was continued until the cell pellet became colorless, and the supernatants were collected in the falcon. Pigment extracts were subjected to evaporation, and the solid fraction was weighed to determine the pigment content (López et al. 2023; Papapostolou et al. 2023).

### 2.3 Molecular Identification of Selected Isolate

The bacterial isolate with the highest pigment‐production potential was subjected to molecular identification using 16S ribosomal RNA (rRNA) gene sequencing. For this purpose, the isolate was cultivated in TSB at 30°C under continuous shaking at 150 rpm for 48 h. Following incubation, cells were harvested by centrifugation, and genomic DNA was extracted from the pellet using a commercial DNA isolation kit (Promega wizardR, A2360) according to the manufacturer's instructions. DNA purity and concentration were evaluated spectrophotometrically at 260 and 280 nm. The 16S rRNA gene was amplified via polymerase chain reaction (PCR) using the primers UNI16S‐F (5′‐ATTCTAGAGTTTGATCATGGCTCA) and UNI16S‐R (5′‐ATGGTACCGTGTGACGGGCGGTGTGTA). The resulting amplicons were ligated into the pGEM‐T vector (Promega, UK), and recombinant plasmids were isolated from selected transformants using Wizard Plus SV Minipreps (Promega, UK). Finally, the inserts were sequenced by Macrogen, and the sequences obtained were aligned and compared with reference sequences available in the GenBank database to establish the isolate's taxonomic identity.

### 2.4 Determination of Chemical Composition of Pigment Extract

To elucidate the chemical composition of the pigment extract, an LC‐Q‐TOF‐MS system (Accurate‐Mass Quadrupole Tıme Of Flıght–Q‐TOF LC/MS) equipped with an accurate‐mass analyzer was employed (Maoka 2023; Kurek et al. 2025). The separation of target compounds, particularly the discrimination of isomeric forms like zeaxanthin, was achieved through a C30 reverse‐phase analytical column. A binary gradient system, comprising acidified water and a methanol–isopropanol blend, was utilized for elution. Compounds were validated by matching their experimentally accurate masses against theoretical values with a precision of < 5 ppm, supplemented by MS/MS fragments analysis. For the prevention of photooxidation and thermal degradation of carotenoids, all analytical procedures were conducted under low‐light and controlled temperatures (Meléndez‐Martínez et al. 2022; Maoka 2023; Kurek et al. 2025).

### 2.5 In Vitro Anticancer Activity of Pigment Extract

The in vitro cytotoxicity of the extract was evaluated using healthy normal human dermal fibroblasts (NHDFs) and five different cancer cell lines, breast cancer (MCF7), lung cancer (A549), neuroblastoma (SH‐SY5Y), liver (HepG2), and cervical (HeLa) cancer cell lines. For cytotoxicity experiments, different concentrations of the extract (7.81–1000 µg/mL) were prepared in 1% dimethyl sulfoxide (DMSO) (final DMSO concentration approximately 0.1%). Cells were incubated at 37°C in a humidified medium containing 5% CO_2_, the medium was changed every 2 days, and passaged until cell density reached 70%–80%. After transferring the cells to 96‐well plates, they were treated with different concentrations of extract solutions and then incubated at 37°C for 24 h. The group without pigment extract was used as a control (only 1% DMSO). Following the incubation, cell viability was analyzed using the water‐soluble tetrazolium salt (WST‐1) test. Optical densities of the groups were measured at 450 nm using an ELISA reader (Gavanji et al. 2023; Albayrak et al. 2026).

### 2.6 Mechanisms Underlying Anticancer Effects of Pigment Extract

#### 2.6.1 Quantitative Real‐Time Polymerase Chain Reaction (qRT‐PCR) Analysis of Expression Levels of Apoptosis and Autophagy‐Related Genes

To clarify the molecular mechanisms underlying the anticancer activity of carotenoid extract, apoptosis‐ and autophagy‐related gene expression profiles were investigated. For this purpose, cancer cell lines were seeded into appropriate culture plates and kept under standard incubation conditions (37°C, 5% CO_2_, humidified atmosphere) until reaching approximately 70%–80% confluency. Cells were then treated with the carotenoid extract at the previously determined IC_50_ values for 24 h (Setiawati et al. 2022; Ladke et al. 2023). In this stage, untreated cells were maintained under identical conditions and were used as the control group. Following treatment, total RNA was isolated using a commercially available RNA extraction kit according to the manufacturer's protocol. RNA purity and concentration were evaluated spectrophotometrically. Complementary DNA (cDNA) was synthesized from equal amounts of RNA using a reverse transcription system.

qRT‐PCR analysis was subsequently performed using gene‐specific primers targeting key regulators of apoptosis (p53, Bax, Caspase‐3, Caspase‐8, Caspase‐9, Bcl‐2, and Akt1) and autophagy (Beclin‐1 and mechanistic target of rapamycin [mTOR]), with a housekeeping gene (β‐actin) employed for normalization. The primer list was provided in Table 1. Amplification reactions were carried out using a SYBR Green‐based detection system under optimized cycling conditions. Relative gene expression levels were calculated using the $2^{-∆∆C_{t}}$ method (Livak and Schmittgen 2001), comparing treated samples to the untreated control group. All experiments were performed in triplicate to ensure reproducibility, and statistical analyses were applied to determine the significance of differences in gene expression, thereby providing insight into the involvement of apoptotic and autophagic pathways in the cellular response to the extract treatment.

**Table 1:** Primer sequences used for quantitative real‐time polymerase chain reaction.

Gene	Primer sequences (5′–3′)	Reference
p53	F: AGAGTCTATAGGCCCACCCC	Ali et al. (2017)
	R: GCTCGACGCTAGGATCTGAC	
Bax	F: CCCCCGAGAGGTCTTTTTCC	Barrios‐Arpi et al. (2022)
	R: CCTTGAGCACCAGTTTGCTG	
Bcl‐2	F: TCTCATGCCAAGGGGGAAAC	Barrios‐Arpi et al. (2022)
	R: TCCCGGTTATCGTACCCTGT	
Caspase‐3	F: GTGGAGGCCGACTTCTTGTA	Barrios‐Arpi et al. (2022)
	R: TTTCAGCATGGCACAAAGCG	
Caspase‐9	F: CTGTCTACGGCACAGATGGAT	Jiao et al. (2020)
	R: GGGACTCGTCTTCAGGGGAA	
Akt1	F: GAAGGACGGGAGCAGGC	Barrios‐Arpi
	R: TGTACTCCCCTCGTTTGTGC	
Caspase‐8	F: TGGTTCATCCAGTCGCTTTG	Jiao et al. (2020)
	R: AATTCTGTTGCCACCTTTCG	
β‐Actin	F: CTGGAACGGTGAAGGTGACA	Jiao et al. (2020)
	R: AAGGAACTTCCTTGAACAATGCA	

#### 2.6.2 Determination of Apoptotic Protein Levels by ELISA

As mentioned above, the cancer cell lines were subjected to the carotenoid extract (at an IC_50_ value) for 24 h (Setiawati et al. 2022; Ladke et al. 2023). At the end of the incubation period, both detached and attached cells were collected to ensure an accurate representation of apoptotic populations. The adherent cells were released using a trypsinization step, neutralized with complete medium, and combined with the floating cells. The pooled cells were washed with ice‐cold phosphate‐buffered saline (PBS) and pelleted by centrifugation.

During the lysis and protein extraction phase, 200 µL lysis buffer (RIPA buffer) containing a protease inhibitor cocktail was added to the cell pellet. The suspension was then incubated on ice for 30 min to ensure complete cell lysis (during this stage, vortexing was performed every 10 min to increase homogenization of the suspension). The protein lysate was centrifuged at high speed (12,000*g*) for 10 min at 4°C to precipitate cell debris. The resulting protein‐rich supernatant was carefully transferred to a new tube. The protein concentration of the supernatant was determined by the bicinchoninic acid method (Smith et al. 1985; Goldring 2019), and appropriate dilutions were performed to ensure all samples had equal total protein content prior to ELISA analysis.

The levels of target proteins Bax (ab199080), Bcl‐2 (ab119506), cleaved caspase‐3 (ab220655), cleaved caspase‐8 (ab315069), and cleaved caspase‐9 (ab119508) were determined using commercial ELISA kits. In each experimental condition, two independent biological replicates were performed, and each lysate was measured in technical triplicate wells. Prior to ELISA, samples were diluted with the kit's sample diluent to fall within the linear range of the assay. A series of the target protein standards was prepared by serial dilution to prepare calibration curves. Subsequently, standards and diluted samples were dispensed in precoated 96‐well microplates. Subsequent incubation, washing, and detection steps were performed according to the manufacturer's instructions. In the final step, tetramethylbenzidine substrate was added and the plate was incubated in the dark until sufficient color development was achieved. The reaction was terminated using the provided stop solution, and the absorbance was measured at 450 nm in a microplate reader. The absorbances were converted to concentrations (ng/mL) according to the standard curve. The levels of target proteins in the treatment and control groups were normalized to the total protein content and expressed as ng/mg protein.

#### 2.6.3 Evaluation of Apoptosis Using Flow Cytometry

The apoptotic effects of pigment extract on cancer cells were evaluated by flow cytometry (Beckman Coulter CytoFLEX) using Annexin V‐FITC and propidium iodide (PI) double staining (ABP Biosciences, USA). In brief, cells were maintained under standard culture conditions (37°C, 5% CO_2_, and humidified atmosphere) and exposed to the extract at their respective IC50 values for 24 h (Setiawati et al. 2022; Ladke et al. 2023). Untreated cells were used as a control.

At the end of the incubation period, both attached and floating cells were harvested together to prevent loss of apoptotic cells. Following collection, cells were pelleted by centrifugation and washed twice with cold‐PBS. Subsequently, the cell pellets were gently resuspended in Annexin V binding buffer to obtain a final density of approximately 1 × 105 cells/mL. For staining, 100 µL of the prepared cell suspension was incubated with Annexin V‐FITC and PI solutions according to the manufacturer's recommendations. The samples were kept in the dark at room temperature for 15 min to allow proper dye binding. After incubation, the binding buffer was added to each sample prior to analysis.

Fluorescence emissions were detected in appropriate channels for FITC and PI, and compensation adjustments were applied using single‐stained controls to minimize spectral overlap. A minimum of 10,000 events per sample was recorded. Cell debris and aggregates were excluded based on forward and side scatter properties, and doublets were discriminated during analysis to ensure accurate evaluation of the single‐cell population.

Data processing and analysis were performed using CytExpert software (Beckman Coulter). Cell populations were categorized into four distinct groups based on fluorescence distribution: Live cells (Annexin V^−^/PI^−^), early apoptotic cells (Annexin V^+^/PI^−^), late apoptotic cells (Annexin V^+^/PI^+^), and necrotic cells (Annexin V^−^/PI^+^). The results were expressed as percentages of total cell populations.

### 2.7 In Silico Analysis of Anticancer Activities of Pigment Extract

#### 2.7.1 Ligand Preparation

The three‐dimensional structures of the reference compounds and test ligands were extracted from the PubChem database in Structure‐Data File format (https://pubchem.ncbi.nlm.nih.gov/). The reference compounds were chosen based on their known binding affinities for the respective target receptors: Capivasertib (PubChem CID: 25227436) served as the positive control for receptor 4GV1 (Akt1), 3OO (PubChem CID: 78673861) as the reference ligand for receptor 4RG2 (p53), and 1XJ (PubChem CID: 253657663) as the reference ligand for receptor 4LVT (Bcl‐2). The test substances included five carotenoid derivatives with the highest content: beta‐carotene (PubChem CID: 5280489), cryptoxanthin (PubChem CID: 5281235), zeaxanthin (PubChem CID: 5280899), astaxanthin (PubChem CID: 5281224), and phytoene (PubChem CID: 11070449). To achieve energetically advantageous conformations, all ligand structures were energy‐minimized using the Merck Molecular Force Field implementation in UCSF Chimera version 1.19.

#### 2.7.2 Receptor Preparation

The target receptors' three‐dimensional crystal structures were retrieved from the Protein Data Bank (https://www.rcsb.org/) using accession numbers 4GV1, 4RG2, and 4LVT. These structures were chosen based on their high‐resolution X‐ray diffraction data and the presence of bound co‐crystallized ligands relevant to the study goals. Protein preparation was carried out using UCSF Chimera version 1.19. All nonstandard residues, such as crystallographic water molecules, ions, cofactors, and binding ligands, were deleted to avoid potential interference with docking calculations. Polar hydrogen atoms were introduced to the protein structures in accordance with standard ionization states at physiological pH (7.4), and all atoms received Gasteiger partial charges. To alleviate steric conflicts, energy was minimized using the conjugate gradient approach with the AMBER ff14SB force field.

#### 2.7.3 Druggability Assessment

The druggability of target binding pockets was assessed using the CavityPlus web server (http://www.pkumdl.cn:8000/cavityplus/#/), which uses the LIGSITE method to detect and characterize protein cavities. This study offered quantitative estimates of pocket druggability scores, hydrophobicity profiles, and volume measurements, allowing for the identification of the most likely binding sites for subsequent molecular docking assessments.

#### 2.7.4 Molecular Docking

PyRx, a computational tool that integrates AutoDock Vina as the docking engine, was used to perform molecular docking (https://pyrx.sourceforge.io/). After merging nonpolar hydrogen atoms and assigning Gasteiger charges, the obtained protein structures were transformed into AutoDock PDBQT format. In a similar manner, ligand structures were created by identifying rotatable bonds and allocating suitable torsion angles. With a grid spacing of 0.375 Å and an exhaustiveness parameter of 8 to guarantee complete conformational sampling, the grid box dimensions were set to cover the full probable binding cavity found during the druggability evaluation. The docking simulation produced up to nine binding poses for each ligand–protein pair. On the basis of the binding affinity (Δ*G*, kcal/mol) and clustering analysis of the resulting poses, the lowest energy conformation was chosen for additional study.

#### 2.7.5 Ligand–Protein Interaction Analysis

The Protein–Ligand Interaction Profiler internet server (https://plip-tool.biotec.tu-dresden.de/plip-web/plip/index) was used to analyze ligand–protein interactions. Hydrogen bonds, hydrophobic contacts, π–π stacking interactions, π–cation interactions, salt bridges, and halogen bonds are among the noncovalent interactions that the server methodically detects and describes. The quality and strength of individual contacts were assessed by extracting interaction parameters like bond distances and angles. To aid in the structural interpretation of the docking results, PyMOL version 2.5 (Schrödinger LLC) and UCSF Chimera were used to create visual representations of ligand–protein complexes, including surface representations and interaction diagrams. To visualize the 2D interaction diagram, BIOVIA Discovery Studio was used.

### 2.8 Statistical Analysis

Three independent experiments were carried out, and each assay was performed in at least two replicates (*n* = 6). Statistical analysis of the data was performed using SPSS 29.0 and GraphPad Prism 8.0 programs. One‐way analysis of variance was employed to compare the differences between groups. Values are expressed as the mean ± standard deviation (mean ± SD). A *p* < 0.05 was considered statistically significant.

## 3 Results and Discussion

### 3.1 Isolation and Screening of Pigment‐Producing Bacteria

Bacteria are known to produce pigments in different colors, such as yellow, orange, red, pink, purple, blue, green, brown, and black (Orlandi et al. 2022; Tang et al. 2025). The literature also reports that soil environments are one of the most important sources for the isolation of pigment‐producing bacteria (Goswami and Bhowal 2014). Therefore, in this study, the isolation of pigment‐producing bacteria was carried out from samples taken from different soil environments (Poplar forest soil, pine forest soil, potting soil, sunflower field soil, potato field soil, and tomato field soil). A total of 50 pigment‐producing bacteria (light yellow, bright yellow, red, and pink color) were isolated from these soil samples on TSA medium. These isolates were then subjected to preliminary examination in terms of colony morphology, pigmentation, and cell morphology, and 15 isolates considered to be distinct were selected.

In the screening step, the ability of 15 isolates to produce pigment in TSB was quantitatively investigated. As seen from the results given in Table 2, among 15 isolates, four isolates (SK2, SK12, SK19, and SK34) produced high amounts of pigment, two isolates (SK7 and SK43) produced moderate amounts, and nine isolates produced low amounts. In particular, the highest total pigment content (128 mg/L) was determined for the isolate SK34, which was obtained from pine forest soil.

**Table 2:** Comparison of pigment production abilities of bacterial isolates.

Isolation source	Isolate code	CDW (g/L)	EA (g/mL)
Tomato field soil	SK2	1.96 ± 0.26^b^	0.109 ± 0.008^ab^
	SK6	1.37 ± 0.14^e^	0.024 ± 0.008^e^
Potato field soil	SK7	1.54 ± 0.18^d^	0.053 ± 0.006^c^
	SK12	1.68 ± 0.12^cd^	0.096 ± 0.012^b^
	SK16	1.54 ± 0.20^d^	0.029 ± 0.006^e^
Sunflower field soil	SK19	1.77 ± 0.22^c^	0.094 ± 0.010^b^
	SK21	2.52 ± 0.32^a^	0.029 ± 0.008^e^
Potting soil	SK23	1.76 ± 0.16^c^	0.025 ± 0.005^e^
	SK29	2.41 ± 0.20^a^	0.031 ± 0.006^de^
Pine forest soil	SK31	1.23 ± 0.24^fg^	0.026 ± 0.005^e^
	SK34	2.16 ± 0.24^ab^	0.128 ± 0.014^a^
	SK38	1.44 ± 0.26^de^	0.042 ± 0.007^d^
Poplar forest soil	SK40	1.12 ± 0.10^g^	0.034 ± 0.006^de^
	SK43	1.81 ± 0.16^c^	0.061 ± 0.008^c^
	SK47	1.87 ± 0.22^bc^	0.027 ± 0.004^e^

*Note:* Three independent experiments were conducted, and measurements were performed at two replicates (*n* = 6). All measurements are presented as mean ± standard deviation (±SD) of six determinations (*n* = 6). The same alphabetical letters in the same column did not show statistically significant differences at the *p* ≤ 0.05 level.

Abbreviations: CDW, cell dry weight; EA, enzyme activity.

### 3.2 Molecular Identification of SK34 Isolate

**Figure 1:**
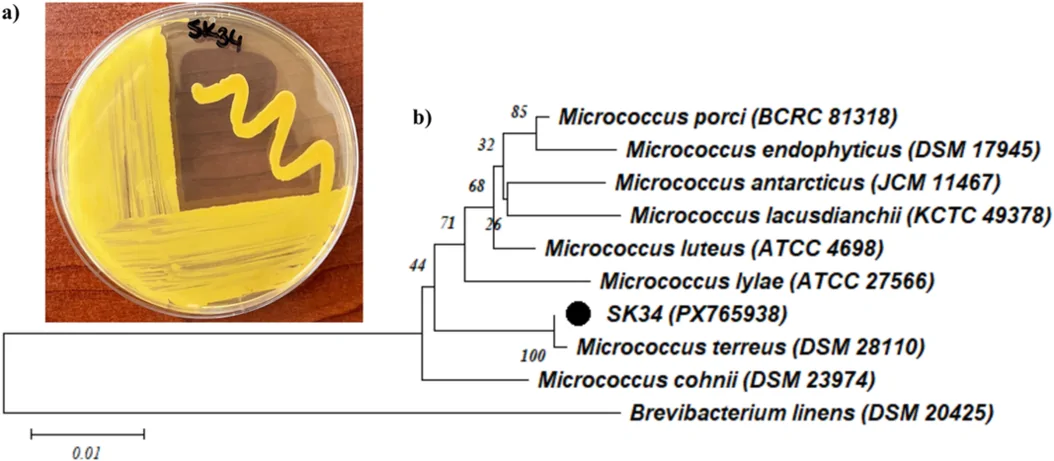
Morphological appearance and phylogenetic analysis of strain SK34. (a) Colony morphology of SK34 cultured on agar medium. (b) Phylogenetic tree based on 16S ribosomal RNA gene sequences showing the relationship of SK34 with closely related *Micrococcus* species. Bootstrap values (%) are indicated at branch nodes, and the scale bar represents nucleotide substitutions per site. ATCC, American Type Culture Collection; BCRC, Bioresource Collection and Research Center; DSM, Leibniz Institute DSMZ ‐ German Collection of Microorganisms and Cell Cultures; JCM, Japan Collection of Microorganisms; KCTC, Korean Collection for Type Cultures.

The isolate SK34 was determined to develop strong yellow colonies on TSA medium (Figure 1a). BLAST‐based evaluation of the 16S rRNA gene sequence of SK34 against the GenBank database demonstrated a high degree of similarity to *Micrococcus* species. A neighbor‐joining tree, which was constructed according to 16S rRNA sequences of representative *Micrococcus* strains, indicates that the isolate SK34 (GenBank: PX765938) was closely related to *M. terreus* DSM 28110 (Figure 1b).

In the literature, the ability of different species of *Micrococcus* genus, especially *M. luteus*, to produce metabolites of biotechnological importance, including pigments, and their bioremediation capabilities have been tested in numerous studies (Sims et al. 1986; Puyen et al. 2012; Umadevi and Krishnaveni 2013; Hegazy et al. 2024). Conversely, studies investigating the chemical composition and biological activities of carotenoids from *M. terreus* remain extremely limited.

### 3.3 Chemical Composition of Pigment Extract From *M. terreus* SK34

Important types of bacteria‐derived carotenoids include α‐carotene, β‐carotene, phytoene, bacteriorubberin, decaprenoxanthin, zeaxanthin, ζ‐carotene, β‐zeacarotene, arthroxanthin, neurosporen, canthaxanthin, astaxanthin, sarcinoxanthin, and lutein (Ram et al. 2020; López et al. 2023; Arslan, Azad, et al. 2025). In the present study, the chemical composition of the pigment extract of *M. terreus* SK34 was determined by LC‐Q‐TOF‐MS. The analysis revealed a complex carotenoid profile characterized by multiple peaks in total ion chromotogram. Major compounds were tentatively identified based on their retention times, accurate‐mass measurements, and characteristic fragmentation patterns. In this way, a total of eight carotenoids could be identified. Phytoene, zeaxanthin, and β‐cryptoxanthin were determined to be the main carotenoids, followed by β‐carotene, astaxanthin, α‐carotene, lycopene, and sarcinaxanthin, respectively (Table 3 and Supporting Information Materials I and II). These data indicate that the pigment extract of *M. terreus* SK34 consists mainly of carotenoid group pigments. It is known that phytoene is colorless, and zeaxanthin is yellow. Considering this knowledge, it was concluded that zeaxanthin with the second highest abundance was the main factor responsible for strong yellow pigmentation of the isolate.

**Table 3:** Chemical composition of carotenoid extract from *Micrococcus terreus*.

Main pigments	Area	Area (%)
Beta‐carotene	1945	10.38
Alpha‐carotene	1501	8.01
Lycopene	1411	7.53
Phytoene	5803	30.97
Beta‐cryptoxanthin	2567	13.70
Zeaxanthin	3205	17.10
Sarcinaxanthin	534	2.85
Astaxanthin	1772	9.46

### 3.4 In Vitro Anticancer Activity of Carotenoid Extract

Previous studies have reported that bacterial carotenoids possess anticancer properties in several cancer models (Rezaeeyan et al. 2017; Jinendiran et al. 2020; Tapia et al. 2021; Yu et al. 2022). Consistent with these reports, the carotenoid extract from *M. terreus* SK34 exhibited potent anticancer activity against multiple cancer cell lines, including HepG2 (liver), A549 (lung), SH‐SY5Y (neuroblastoma), MCF‐7 (breast), and HeLa (cervix) cancer cell lines (*p* < 0.05–*p* < 0.0001). The extract was able to kill cancerous cells even at low concentrations, and its anticancer effect increased with increasing concentrations, indicating that the extract inhibited cancer cell viability in a dose‐dependent manner.

**Figure 2:**
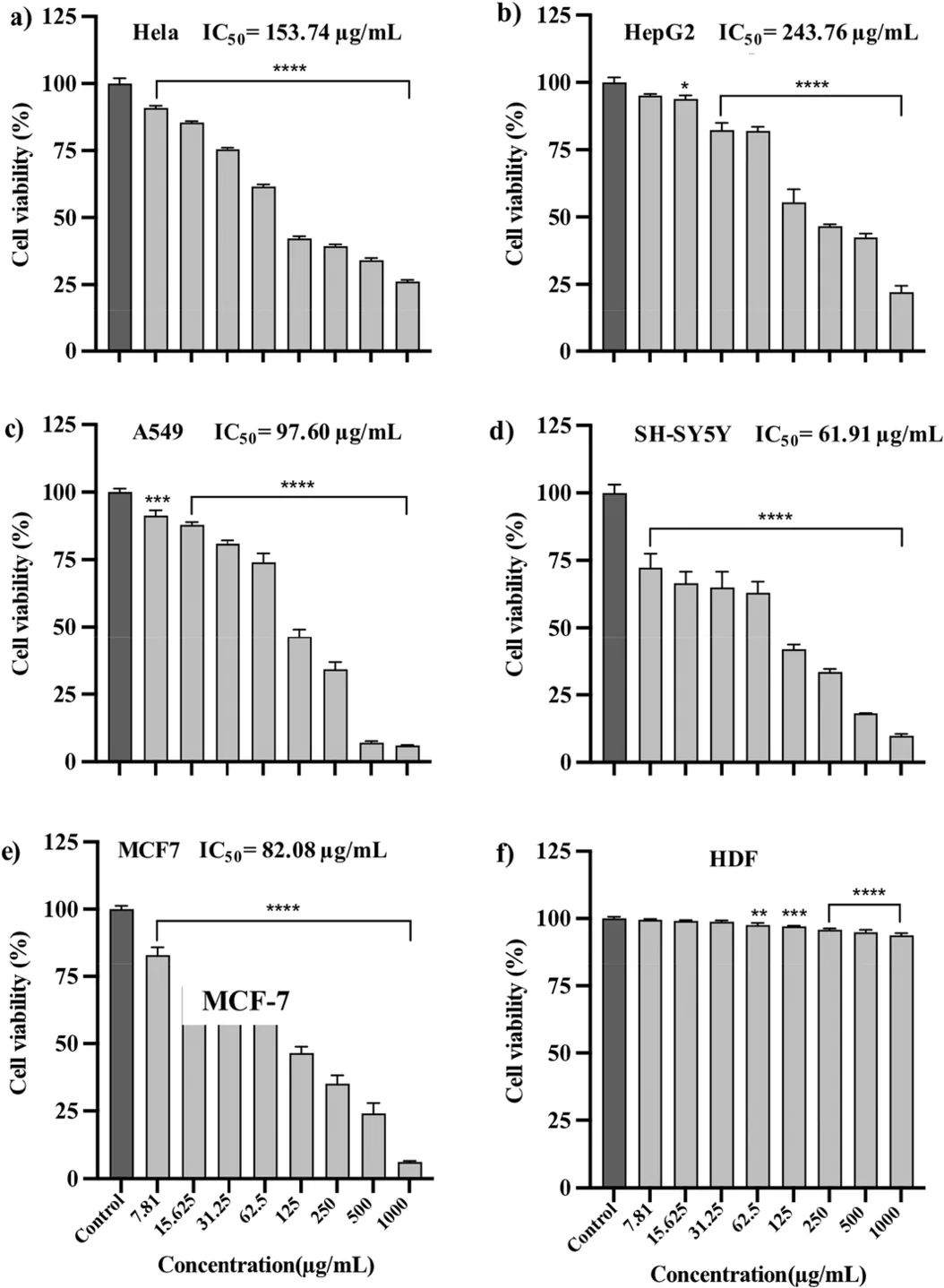
Cytotoxic effects of carotenoid extract on different cell lines. The carotenoid extract was applied to the cells at different concentrations for 24 h. HepG2 (liver), A549 (lung), SH‐SY5Y (neuroblastoma), MCF‐7 (breast), HeLa (cervix) cancer cell lines, and HDF (healthy human dermal fibroblasts). Two independent experiments were conducted and measurements were made in at least three replicates (*n* = 6). *p* > 0.05 (ns, not significant), *p < 0.05 (significant), **p < 0.01 (highly significant), ***p < 0.001 (very highly significant), and ****p < 0.0001 (extremely significant).

The IC_50_ values of extract against HeLa, HepG2, A549, SH‐SY5Y, and MCF7 were determined to be 153.74, 243.76, 97.60, 61.91, and 82.08 μg/mL, respectively (Figure 2a–e). These IC_50_ values are lower than or close to the IC_50_ values of carotenoid extracts tested in earlier works (Rezaeeyan et al. 2017; Zohari et al. 2021; Giani et al. 2023; Wei et al. 2023; Abdelazim et al. 2025). These results indicated that the extract exerted the strongest anticancer efficacy against SH‐SY5Y (Figure 2d), followed by MCF‐7 cell line (Figure 2e).

A key desirable characteristic of anticancer molecules is that they do not cause toxicity in healthy cells (Ioele et al. 2022; Blagosklonny 2023). As seen from Figure 2f, the extract exhibited lower cytotoxicity in healthy human dermal fibroblasts as opposed to cancer cell lines. For example, cell viability in NHDF cells remained above 90% even at the highest concentration tested (1000 µg/mL). Whereas a significant reduction in viability was observed in cancer cell lines under the same conditions. Since the IC_50_ value for NHDF could not be determined within the tested concentration range, selectivity index (SI) values were presented as minimum estimates (SI > X) based on the highest tested concentration (1000 µg/mL). For example, minimum SI was estimated to be greater than 4.1 for HepG2 (1000:243.76), 6.5 for HeLa (1000:153.74), 10.3 for A549 (1000:97.6), 12.1 for Mcf7 (1000:82.08), and 16.1 for SH‐SY5Y (1000:61.91). The literature reports that SI greater than 2 is generally considered indicative of selective cytotoxicity, while values above 10 reflect high selectivity (Lopez‐Lazaro 2015; Lima et al. 2025). In this study, SI values greater than 4 were calculated for all cancer cell lines. Furthermore, SI values above 10 were calculated for A549, Mcf7, and SH‐SY5Y cancer cell lines. Overall, these results indicate a clear and pronounced selective cytotoxic effect of the extract toward cancer cells.

### 3.5 Mechanisms Underlying Anticancer Effects of Pigment Extract

Apoptosis, also known as programmed cell death, is the most studied mechanism in cancer biology. This mechanism is maintained by the balance in activities of proapoptotic and antiapoptotic genes (Fiandalo and Kyprianou 2012; Pistritto et al. 2016; Chaudhry et al. 2022; Sousa et al. 2022). There is a balance in the functioning of proapoptotic and apoptotic genes in healthy cells. Disruptions in the functioning of these genes lead to uncontrolled cell division and the development of cancer (Fiandalo and Kyprianou 2012; Pistritto et al. 2016; Mustafa et al. 2024). Accordingly, there is an increasing interest in anticancer therapies, which focus on triggering apoptosis through the modulation of the activities of apoptosis‐related genes, that is, the upregulation of proapoptotic genes and the downregulation of antiapoptotic genes (Mustafa et al. 2024; Mosadegh et al. 2025).

Autophagy is another important mechanism playing a critical role in cancer biology. This mechanism, which relies on the cell digesting and eliminating damaged or overloaded components within itself, hence helps the cell survive and maintain homeostasis (Glick et al. 2010; Yun and Lee 2018; Jalali et al. 2025). However, when cells encounter excessive stress, autophagy becomes overactivated, leading to cell death. For example, a significant increase in the level of Beclin‐1, along with the reduction in mTOR level, is reported to trigger autophagy. Therefore, triggering autophagy is seen as another promising approach in cancer treatments (Yun and Lee 2018; Jalali et al. 2025). Considering this knowledge in the literature, the anticancer efficacy of carotenoid extract was investigated in the context of apoptosis and autophagy mechanisms.

### 3.6 Investigation of Apoptotic and Autophagic Mechanisms via qRT‐PCR Analysis

To determine the underlying mechanisms of the anticancer effects of the carotenoid extract, the messenger RNA levels of five proapoptotic genes (p53, Bax, Casp3, Casp8, and Casp9), two antiapoptotic genes (Bcl‐2 and Akt1), and two autophagy‐related genes (Beclin‐1 and mTOR) were analyzed by quantitative polymerase chain reaction (qPCR). The extract (at IC_50_ value) was applied to the SH‐SY5Y cell line, in which the highest cytotoxic effect was determined.

**Figure 3:**
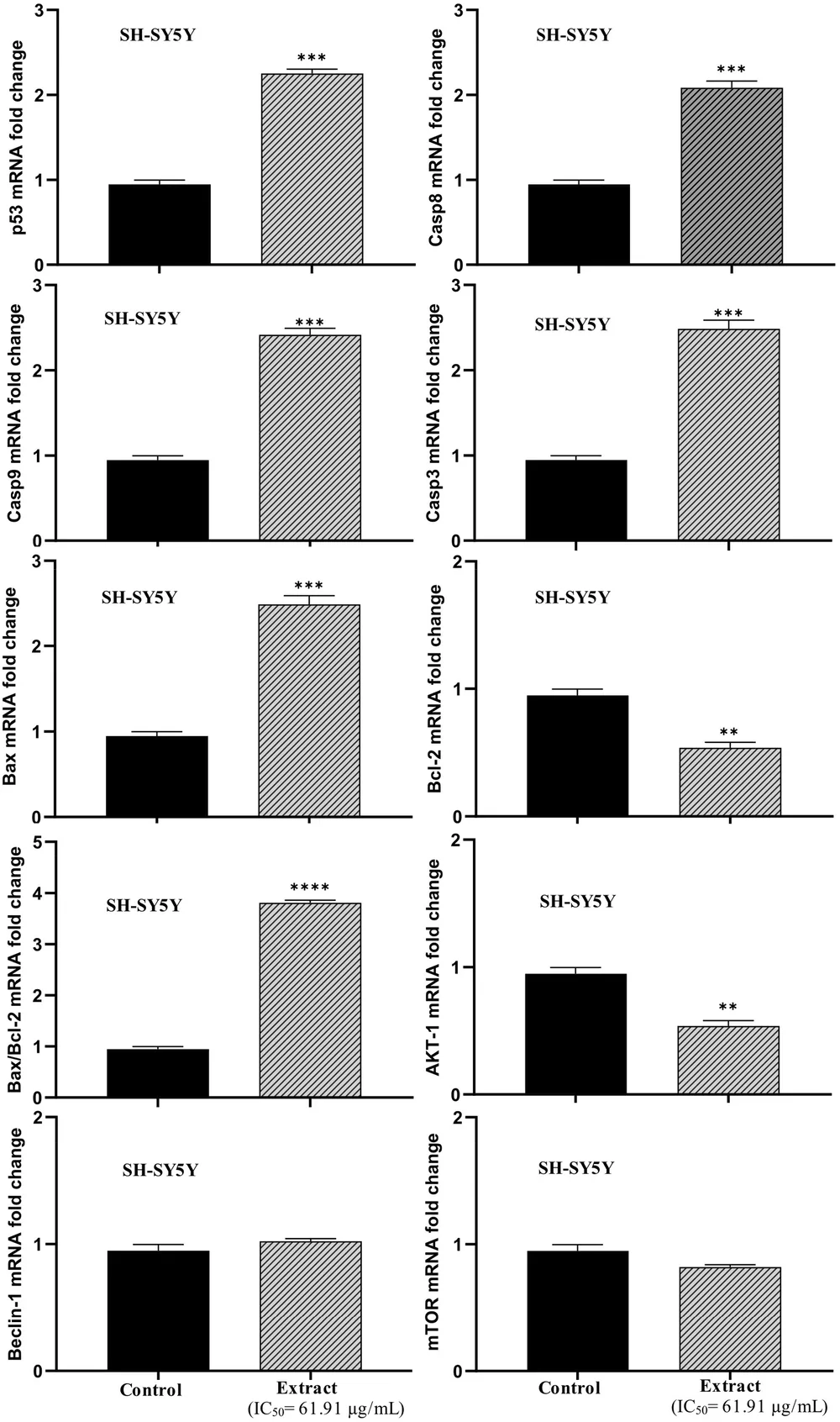
Effect of carotenoid extract on messenger RNA (mRNA) levels of apoptosis and autophagy‐related genes in SH‐SY5Y cancer cell line. The carotenoid extract was applied to the cells at IC_50_ for 24 h. Two independent experiments were conducted, and measurements were made in at least three replicates (*n* = 6). *p* > 0.05 (ns, not significant), *p < 0.05 (significant), **p < 0.01 (highly significant), ***p < 0.001 (very highly significant), and ****p < 0.0001 (extremely significant).

The results revealed that, compared with the control, the extract caused statistically significant increases in the expression levels of p53, Bax, Casp3, Casp8, and Casp9, along with reductions in the expression levels of Bcl‐2 and Akt1 (Figure 3a–f). Specifically, the Bax/Bcl‐2 ratio was determined to exhibit a marked increase in the extract‐treated cells (Bax/Bcl‐2 ratio: 3.85) when compared with untreated control cells. These results elucidated that treatment of SH‐SY5Y cells with the carotenoid extract induced a pronounced upregulation of apoptosis‐related genes as compared with untreated controls.

Casp8 and Casp9 are initiator caspases that play a central role in apoptotic signaling pathways in cells. The former activates death receptors on the cell surface, triggering the extrinsic pathway, while the latter activates the intrinsic (mitochondrial) pathway by causing DNA damage, oxidative stress, or damage to the mitochondrial membrane structure. Ultimately, both genes trigger apoptosis by activating the executioner Casp3 (Singh and Lim 2022; Sahoo et al. 2023). In this study, the gene expression analysis also revealed that the extract caused the concurrent increases in initiator Casp8 (Figure 3b) and Casp9 (Figure 3c) levels, together with the executioner Casp3 level (Figure 3d). These increases suggested that the carotenoid extract may activate both extrinsic and intrinsic apoptotic pathways. However, the greater change in caspase‐9 expression level suggests that the extract may stimulate the intrinsic pathway more.

Gene expression analyses demonstrated that the extract caused small changes in the expression levels of autophagy‐related genes Beclin‐1 and mTOR in the treatment group compared with the control. Namely, there was a small increase in Beclin‐1 level, along with a small decrease in mTOR level. However, these alterations in Beclin‐1 and mTOR levels were not statistically significant. Overall, the results from gene expression analyses suggest that the carotenoid extract may exert anticancer effects primarily through promoting apoptosis rather than autophagy.

### 3.7 ELISA‐Based Analysis of Apoptosis‐Related Proteins

**Figure 4:**
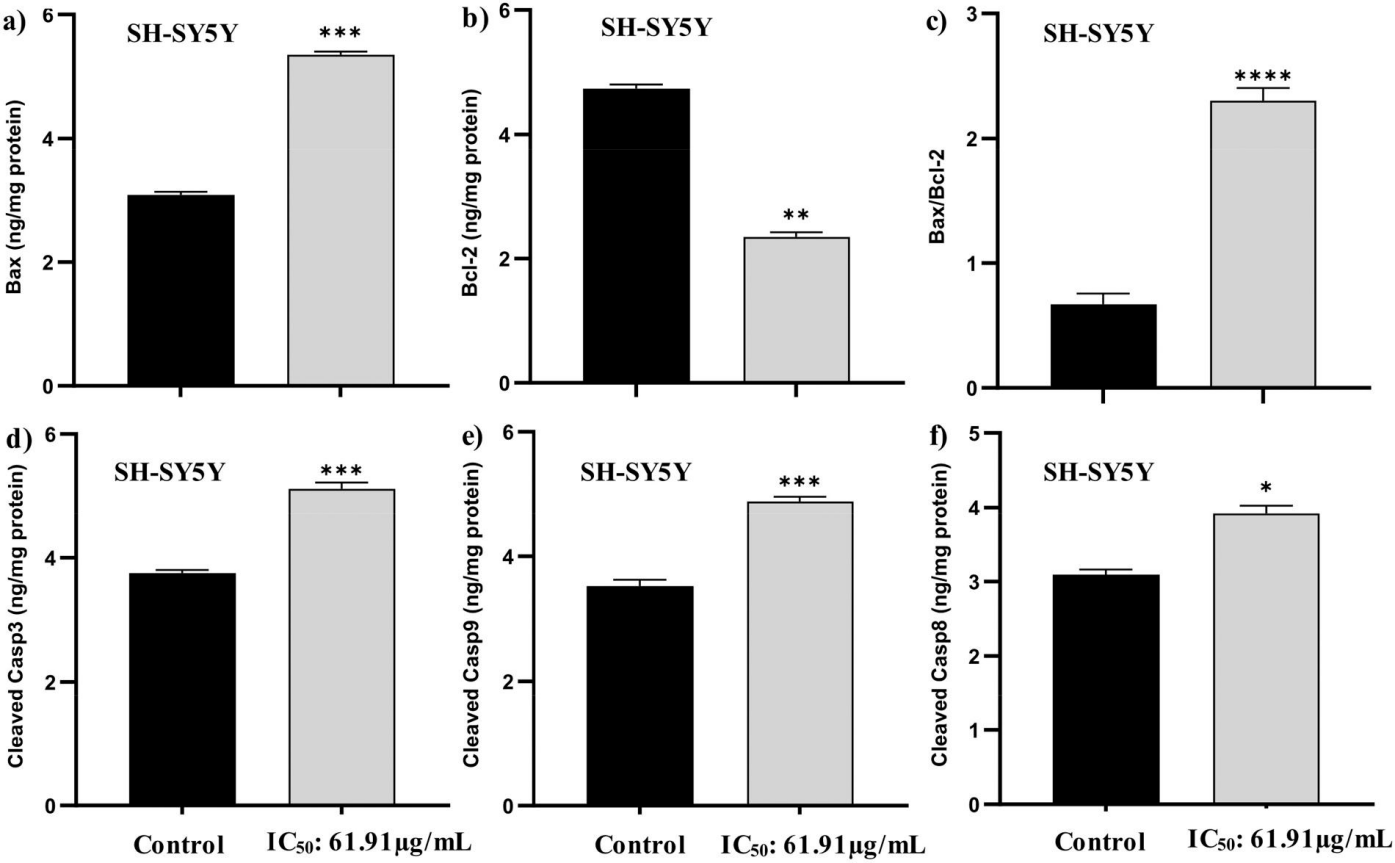
ELISA‐based analysis of apoptosis‐related proteins. The control group received no treatment. (a) Bax/Bcl‐2 ratio, (b) Bax protein levels, (c) Bcl‐2 protein levels, (d) cleaved caspase‐3, (e) cleaved caspase‐9, and (f) cleaved caspase‐8. Protein levels are expressed as ng/mg protein. Data are presented as mean ± standard deviation (SD). Statistical significance was determined by comparison with the control group. *p < 0.05; **p < 0.01; ***p < 0.001; ****p < 0.0001.

ELISA analysis demonstrated that the extract treatment significantly upregulated Bax protein levels (*p* < 0.001) (Figure 4a) alongside a decrease in Bcl‐2 level (*p* < 0.01) (Figure 4b), thereby causing a marked increase in Bax/Bcl‐2 ratio (*p* < 0.0001) (Figure 4c). Furthermore, cleaved caspase‐3 (Figure 4d) and cleaved caspase‐9 levels (Figure 4e) were both significantly elevated (*p* < 0.001) in the treated group relative to the untreated control group, indicating activation of the apoptotic cascade. Cleaved caspase‐8 also showed a statistically significant but less pronounced increase (*p* < 0.05) in the treated group in comparison to the untreated control group (Figure 4f). In summary, the results from ELISA analysis support the gene expression results from qPCR analysis, namely, indicating that the extract can induce apoptosis in the SH‐SY5Y cell line by increasing the Bax/Bcl‐2 ratio and activating caspases (especially caspase‐9 in the intrinsic apoptotic pathway).

### 3.8 Evaluation of Apoptosis by Flow Cytometry

**Figure 5:**
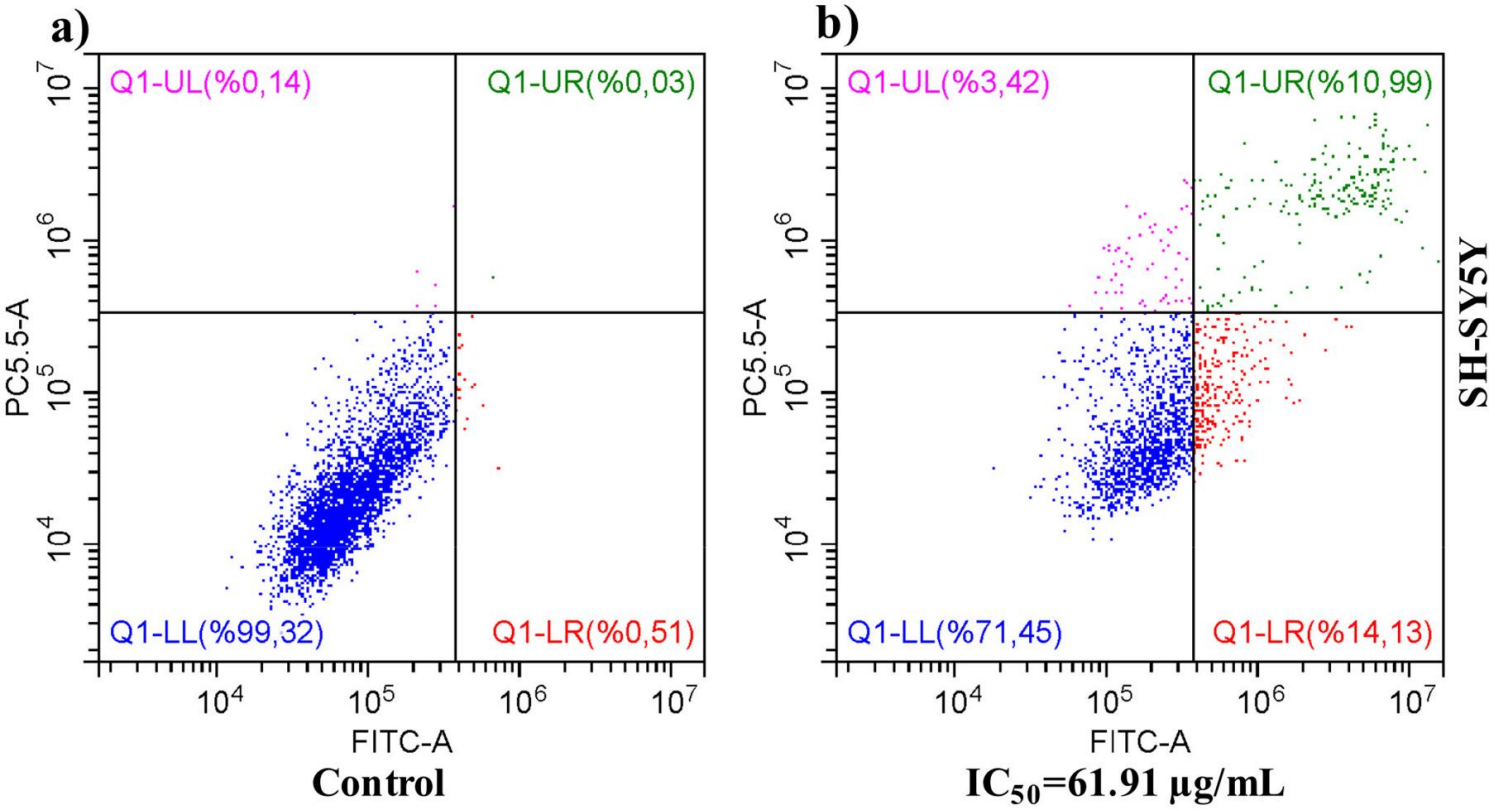
Flow cytometric analysis of apoptosis in SH‐SY5Y cells. (a) The control group received no treatment and (b) the carotenoid extract was applied to the cells at IC_50_ for 24 h. Cells were stained with Annexin V‐FITC (*x*‐axis) and PI (*y*‐axis). Quadrants indicate viable (Q1‐LL), early apoptotic (Q1‐LR), late apoptotic (Q1‐UR), and necrotic (Q1‐UL) cells. Treatment increased apoptotic cell populations compared with the control.

The results from flow cytometry analysis demonstrated that the extract exerted a significant apoptotic effect in the SH‐SY5Y cell line (Figure 8). While the majority of cells in the control group were viable (Q1‐LL: 92.32%), the rates of early apoptosis (Q1‐LR: 0.51%) and late apoptosis/secondary necrosis (Q1‐UR: 0.03%) were extremely low (Figure 5a). In the SH‐SY5Y cell line treated with the extract at the IC_50_ dose, the viable cell rate decreased to 71.45%, while the rate of early apoptotic cells increased to 14.13%, the rate of late apoptotic/secondary necrosis cells increased to 10.99%, and the total apoptosis rate increased to 25.12% (Figure 5b). This finding is particularly relevant for anticancer therapy, as effective chemotherapeutic agents are expected to induce controlled cell death, progressing from early to late apoptosis (Alshehade et al. 2024; Hassan and Ahmad 2026). Overall, these results indicate that the extract may induce cell death mainly through apoptosis.

### 3.9 In Silico Analysis of Anticancer Efficacy of Carotenoid Extract

In vitro experiments and gene expression analyses revealed that the carotenoid extract exhibited a more pronounced cytotoxic effect on two cancer lines. Gene expression, protein assay (ELISA), and flow cytometry analyses suggested that apoptosis was the primary mechanism underlying the anticancer activity of the carotenoid extract. To support this finding, the potential anticancer effects of five major compounds in carotenoid extract were also investigated using a molecular docking approach via apoptosis‐related target genes.

Molecular docking for receptor 4GV1 (Akt1) was carried out using a grid box with dimensions of 47.78 × 51.05 × 62.34 Å, centered at coordinates (−26.5269, 2.6601, 16.0403), with an exhaustiveness setting of 8. β‐carotene (CID: 5280489) had the highest affinity at −9.0 kcal/mol, followed by cryptoxanthin (CID: 5281235) at −8.7 kcal/mol, the reference compound capivasertib (CID: 8.6 kcal/mol), zeaxanthin (CID: 5280899) and astaxanthin (CID: 5281224) at −8.5 kcal/mol, and phytoene (CID: 11070449). When compared with the corresponding lowest‐energy conformations, all docked poses had root‐mean‐square deviation (RMSD) values of 0.0 Å, demonstrating convergence to stable binding modes within the active region of the receptor.

**Figure 6:**
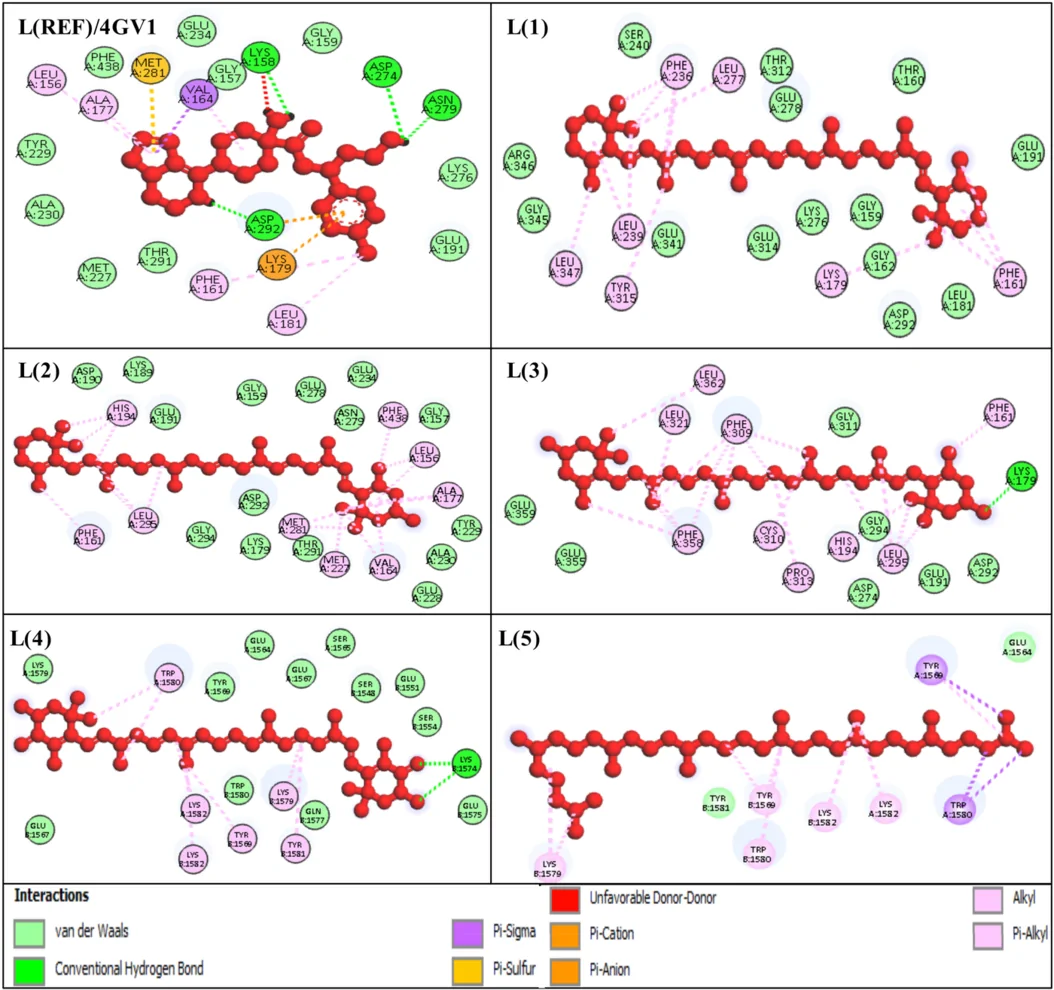
Molecular docking analysis of the ligand interactions with AKT1 (PDB: 4GV1). The ligands were β‐carotene (L1), cryptoxanthin (L2), zeaxanthin (L3), astaxanthin (L4), phytoene (L5), and the reference ligand capivasertib (LREF). PDB, Protein Data Bank.

The molecular interaction profiles of the 4GV1 target protein with the reference ligand capivasertib L(REF) and five different carotenoid compounds β‐carotene (L1), cryptoxanthin (L2), zeaxanthin (L3), astaxanthin (L4), and phytoene (L5) as determined by structural analysis are shown in Figure 6. The experimental ligands show a clear preference for hydrophobic stabilization, which is primarily mediated by amino acid residues like PHE 161 and LEU 193 in all complexes, whereas the reference ligand engages the binding site via a cohesive network of three hydrogen bonds with residues ALA 158, LYS 276, and ASN 279, along with a salt bridge formation with ASP 203. Interestingly, only astaxanthin (L4) exhibits a hydrogen bonding connection with residue LYS 179 among the examined chemicals; in contrast, van der Waals forces and hydrophobic contacts completely control the interactions for β‐Carotene, cryptoxanthin, and phytoene. These different binding signatures imply that although the carotenoids share the same basic pocket, their different binding affinities and inhibitory potential against the 4GV1 target may be greatly impacted by their lack of broad polar connectivity in comparison to the reference ligand.

Molecular docking for receptor 4RG2 (p53) was performed using a grid box with dimensions of 53.60 × 51.35 × 44.82 Å and an exhaustiveness value of 8, centered at coordinates (−16.8455, −13.9962, −11.9601). The carotenoid derivatives displayed moderate to good binding energies, with the reference molecule 3OO (CID: 78673861) showing the maximum binding affinity at −8.7 kcal/mol: Astaxanthin (CID: 5281224) attained −7.8 kcal/mol, cryptoxanthin (CID: 5281235) at −7.7 kcal/mol, zeaxanthin (CID: 5280899) at −7.5 kcal/mol, phytoene (CID: 11070449) at −4.6 kcal/mol, and β‐carotene (CID: 5280489). The top‐ranked docking poses exhibited RMSD values of 0.0 Å, indicating consistent convergence of the docking algorithm toward a single binding orientation within the receptor binding pocket. This finding supports the reproducibility of the predicted binding pose under the applied docking procedure, although RMSD alone does not necessarily indicate binding stability, which requires further validation by molecular dynamics simulations (Ferreira et al. 2015; Pagadala et al. 2017).

**Figure 7:**
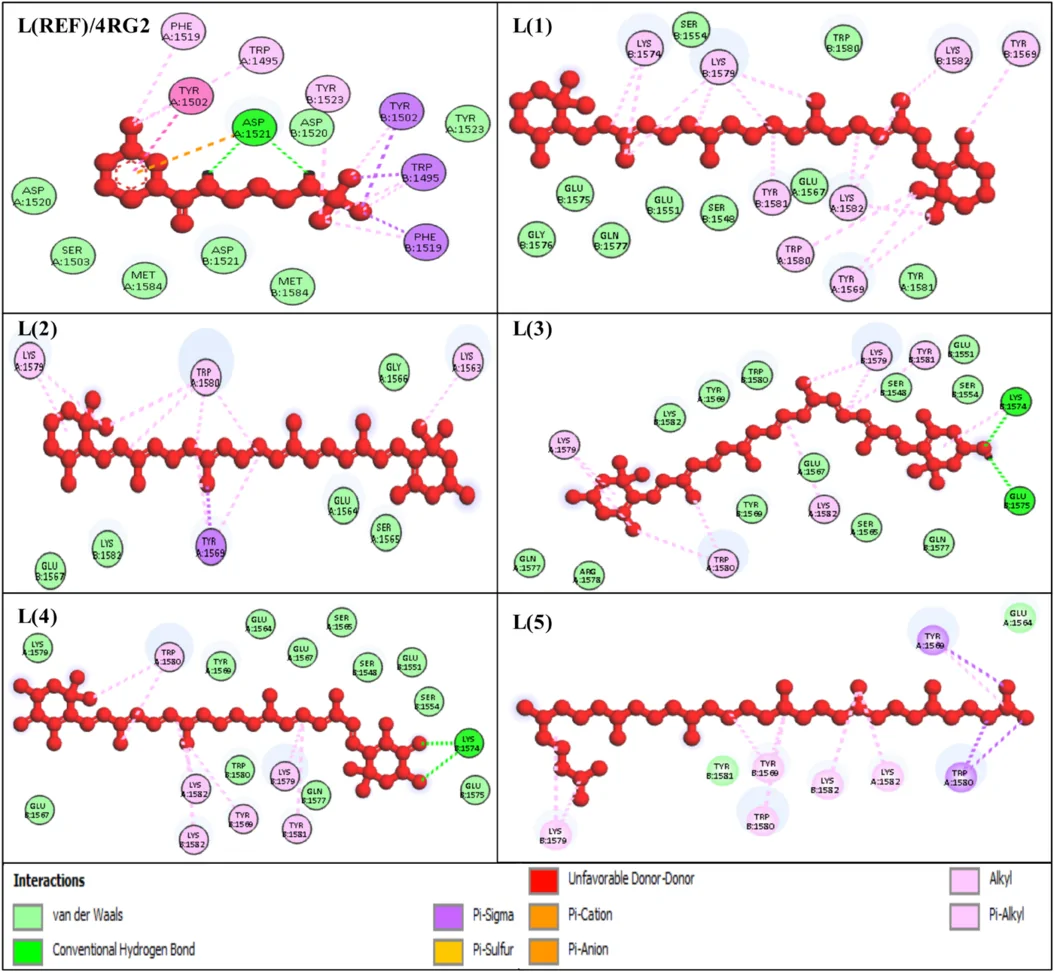
Molecular docking analysis of the ligand interactions with p53 (PDB: 4RG2). The ligands were β‐carotene (L1), cryptoxanthin (L2), zeaxanthin (L3), astaxanthin (L4), phytoene (L5), and the reference ligand 3OO (LREF). PDB, Protein Data Bank.

The comparative molecular docking profiles of the 4RG2 target protein interacting with five carotenoid derivatives (L1–L5) and the reference ligand 3OO (LREF) are shown in Figure 7, which clearly shows a divergence in stabilization mechanisms. The evaluated carotenoid complexes are primarily controlled by vast networks of hydrophobic interactions, which consistently engage important aromatic and aliphatic residues like TYR 1569 A, PHE 1519B, and TRP 1580 A across all five systems, whereas the reference ligand forms a single, strong hydrogen bond with residue ASP 1521 A. Interestingly, only zeaxanthin (L3) and astaxanthin (L4) show polar anchoring via hydrogen bonds with LYS 1574B and SER 1546B/TRP 1580 A, respectively, while β‐carotene (L1), cryptoxanthin (L2), and phytoene (L5) only use van der Waals contacts. In summary, oxygenated carotenoids, namely, astaxanthin and zeaxanthin, were determined to display favorable binding affinities toward p53. Although the calculated binding energies were lower than those obtained for the native ligand, the interaction profiles revealed that these carotenoids established both hydrophobic contacts and hydrogen‐bond interactions with residues located within the binding pocket. Such interaction patterns may contribute to stabilization of ligand–protein complexes and could potentially influence p53‐associated signaling pathway (Milani et al. 2017; Shoaib et al. 2023). However, it should be noted that molecular docking predicts the thermodynamic feasibility of ligand binding rather than direct activation of protein function. Consequently, the biological significance of these interactions should be interpreted together with the experimental findings rather than on the basis of docking scores alone (Ferreira et al. 2015; Pagadala et al. 2017).

Molecular docking was carried out for receptor 4LVT (Bcl‐2) utilizing a grid box with dimensions of 51.85 × 71.62 × 62.96 Å and an exhaustiveness setting of 8, centered at coordinates (10.1501, 16.2008, 14.9505). Zeaxanthin (CID: 5280899) had the highest affinity among the test compounds at −9.1 kcal/mol, followed by astaxanthin (CID: 5281224) at −9.0 kcal/mol, β‐carotene (CID: 5280489) and cryptoxanthin (CID: 5281235) at −8.9 kcal/mol, and phytoene (CID: 11070449) at −6.8 kcal/mol. Stable and repeatable binding conformations within the receptor's active region were confirmed by the convergence of all docked poses with RMSD values of 0.0 Å.

**Figure 8:**
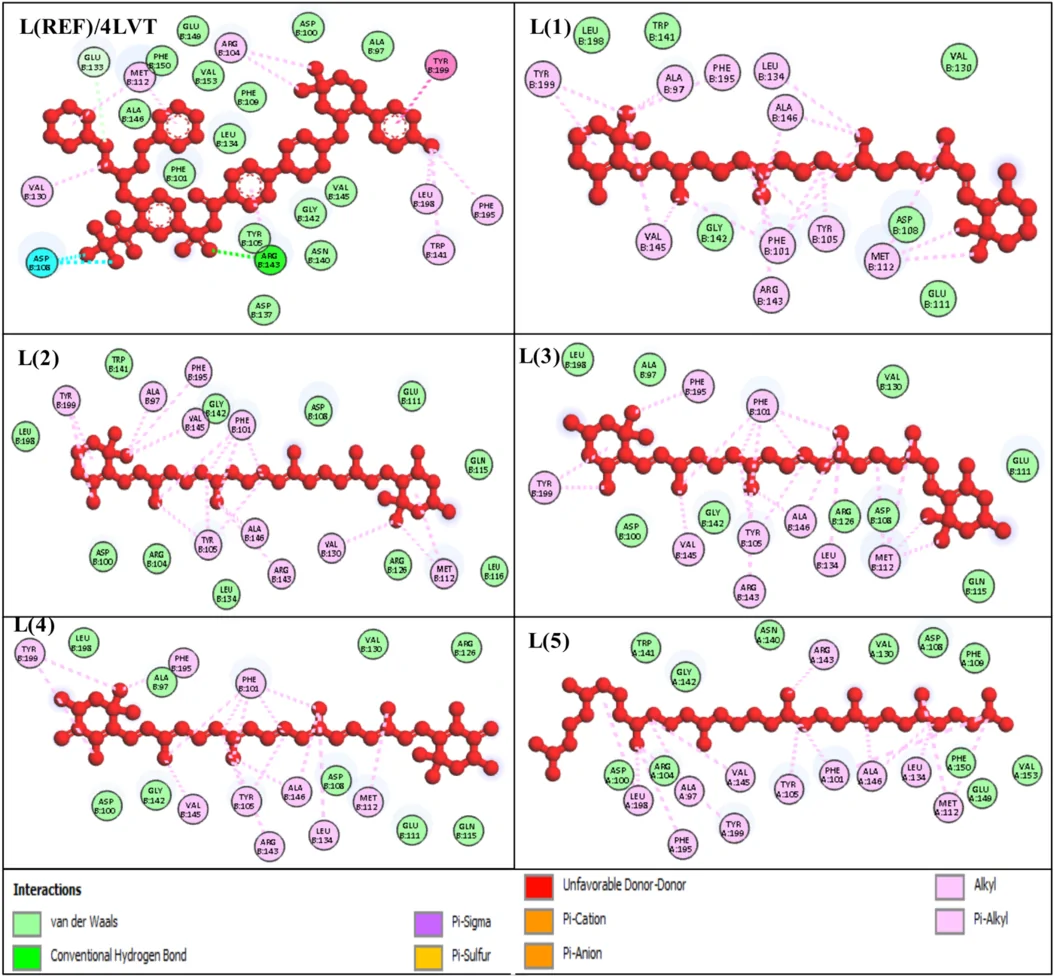
Molecular docking analysis of the ligand interactions with Bcl‐2 (PDB: 4LVT). The ligands were β‐carotene (L1), cryptoxanthin (L2), zeaxanthin (L3), astaxanthin (L4), phytoene (L5), and the reference ligand 1XJ (LREF). PDB, Protein Data Bank.

The results summarized in Figure 8 compare the molecular interactions of the 4LVT target protein with the reference ligand 1XJ L(REF) and five carotenoid variants (L1–L5). All five carotenoid derivatives only interact with the 4LVT binding pocket through hydrophobic and van der Waals forces, in sharp contrast to the reference ligand, which creates a multifaceted stabilization network consisting of two strong hydrogen bonds (ASP 109B and ARG 143B), a halogen bond (PHE 195B), and a salt bridge (GLU 133B). Particularly, the conserved participation of PHE 101B, TYR 105B, and VAL 145B in almost all ligand–protein complexes indicates that these residues constitute an important hydrophobic core involved in carotenoid recognition. Since carotenoids are highly lipophilic molecules characterized by long conjugated polyene chains, hydrophobic packing within nonpolar regions of the Bcl‐2 binding pocket is not unexpected and is fully consistent with their physicochemical properties. Similar interaction profiles have previously been reported for highly hydrophobic natural products targeting antiapoptotic proteins, where shape complementarity and van der Waals interactions contribute substantially to ligand stabilization despite the absence of extensive electrostatic contacts (Ferreira et al. 2015; Abdelazim et al. 2025; Varghese and Ramamoorthy 2025).

The molecular docking analysis demonstrated that major carotenoids in the extract also exhibited favorable binding affinities toward Akt1, p53, and Bcl‐2, suggesting that these natural metabolites may simultaneously regulate multiple checkpoints involved in apoptosis. This multitarget interaction profile is particularly important because apoptosis is orchestrated through highly interconnected signaling networks rather than isolated molecular events. Consequently, compounds capable of modulating several apoptosis‐related proteins often exhibit greater therapeutic potential than agents acting through a single target (Bohn et al. 2021; Baeza‐Morales et al. 2024).

Overall, molecular docking data, consistent with gene expression, ELISA, and flow cytometry findings, suggest that some components of the carotenoid extract may act as an inhibitor of cell survival‐related proteins, particularly Bcl‐2 and AKT1, and activate proapoptotic proteins (p53, Bax, Caspases). These findings are consistent with the fact that the apoptosis‐mediated cell death mechanism is a cornerstone of anticancer actions of carotenoids (Kim et al. 2020; Kacar et al. 2022; Baeza‐Morales et al. 2024). However, further in vivo validation of these preliminary findings is needed to obtain definitive conclusions.

An important aspect of the present findings is that the biological activity of the pigment extract is unlikely to be attributed to a single carotenoid. Instead, the coexistence of multiple carotenoids may generate additive or synergistic biological effects. This is because different carotenoids can influence multiple molecular targets simultaneously. Namely, carotenoid mixtures may exhibit stronger biological activities, for example, anticancer action, than individual purified components (Linnewiel‐Hermoni et al. 2015; Bohn et al. 2021; Islam et al. 2024; Al‐Monofy et al. 2025; Arslan, Azad, et al. 2025). This concept is particularly relevant for cancer therapy, where the coordinated modulation of several signaling pathways is generally more effective than targeting a single molecular mechanism (Linnewiel‐Hermoni et al. 2015; Sui et al. 2024). Consequently, the complex carotenoid composition identified in *M. terreus* SK34 may partly explain the pronounced anticancer activity observed in this study.

Although mechanistic analyses were performed using the IC_50_ concentration of the carotenoid‐rich extract to ensure robust biological responses, it should be acknowledged that some of the observed transcriptional and protein alterations may represent downstream consequences of apoptosis rather than the earliest molecular events responsible for cytotoxicity. Therefore, future studies evaluating sub‐IC_50_ concentrations with time‐course analyses will be valuable to distinguish primary signaling events from secondary apoptotic responses and to further clarify the sequence of molecular mechanisms underlying the anticancer activity of the extract.

## 4 Conclusions

This study provides the first characterization of the carotenoid extract from *M. terreus* SK34, highlighting its significant potential as an anticancer agent. LC‐Q‐TOF‐MS analysis successfully identified eight distinct carotenoids in the extract. The in vitro assays revealed that the extract displayed a remarkable cytotoxicity against multiple human cancer cell lines, most notably neuroblastoma cancer, while maintaining a safe profile toward healthy cells. The qPCR, ELISA, flow cytometry, and docking analyses suggested that the anticancer action is primarily driven by the upregulation of apoptotic pathways. Docking analysis also suggested that four major components of the extract, namely, β‐carotene, astaxanthin, cryptoxanthin, and zeaxanthin, may be the key factors of apoptosis‐mediated anticancer effects. Overall, the carotenoids from *M. terreus* SK34 emerge as promising candidates for integration into the pharmaceutical industry, offering a natural and effective alternative for therapeutic applications. However, there are further targeted studies to validate the extract's mechanisms of action and potential applications in industries.

## Author Contributions

**Sinem Ilayda Karaagac:** data curation, investigation, methodology. **Seyda Albayrak:** methodology, investigation. **Azizeh ShadiDizaji:** investigation, methodology. **Muhammet Enes Kiziler:** methodology, investigation. **Elvan Eroglu:** methodology, investigation. **Nazli Pinar Arslan:** methodology, investigation. **Mesut Taskin:** investigation, supervision, data curation, writing – original draft, writing – review and editing.

## Funding

The authors have nothing to report.

## Ethics Statement

This study does not contain any experiments with human participants or animals.

## Conflicts of Interest

The authors declare no conflicts of interest.

## Supporting information


Supporting File 1

Supporting File 2

## Data Availability

The data that support the findings of this study are available in the Supporting Information Material of this article. The data used to support the findings of this study are included within the main manuscript, Supporting Information Material I (LC‐Q‐TOF‐MS I), and Supporting Information Material II (LC‐Q‐TOF‐MS II).
