# Supplementary material for: Carotenoid‐Rich Pigment Extract From Micrococcus terreus SK34 Induces Apoptosis in SH‐SY5Y Neuroblastoma Cells: Integrated Metabolomic, Molecular Docking, and Experimental Validation
Source: Microbiologyopen. 2026 Sep 25;15(5):e70382. doi: 10.1002/mbo3.70382 (PMC13613168; doi:10.1002/mbo3.70382)
Supplement: Supplementary file 1 [file MBO3-15-e70382-s001.pdf]

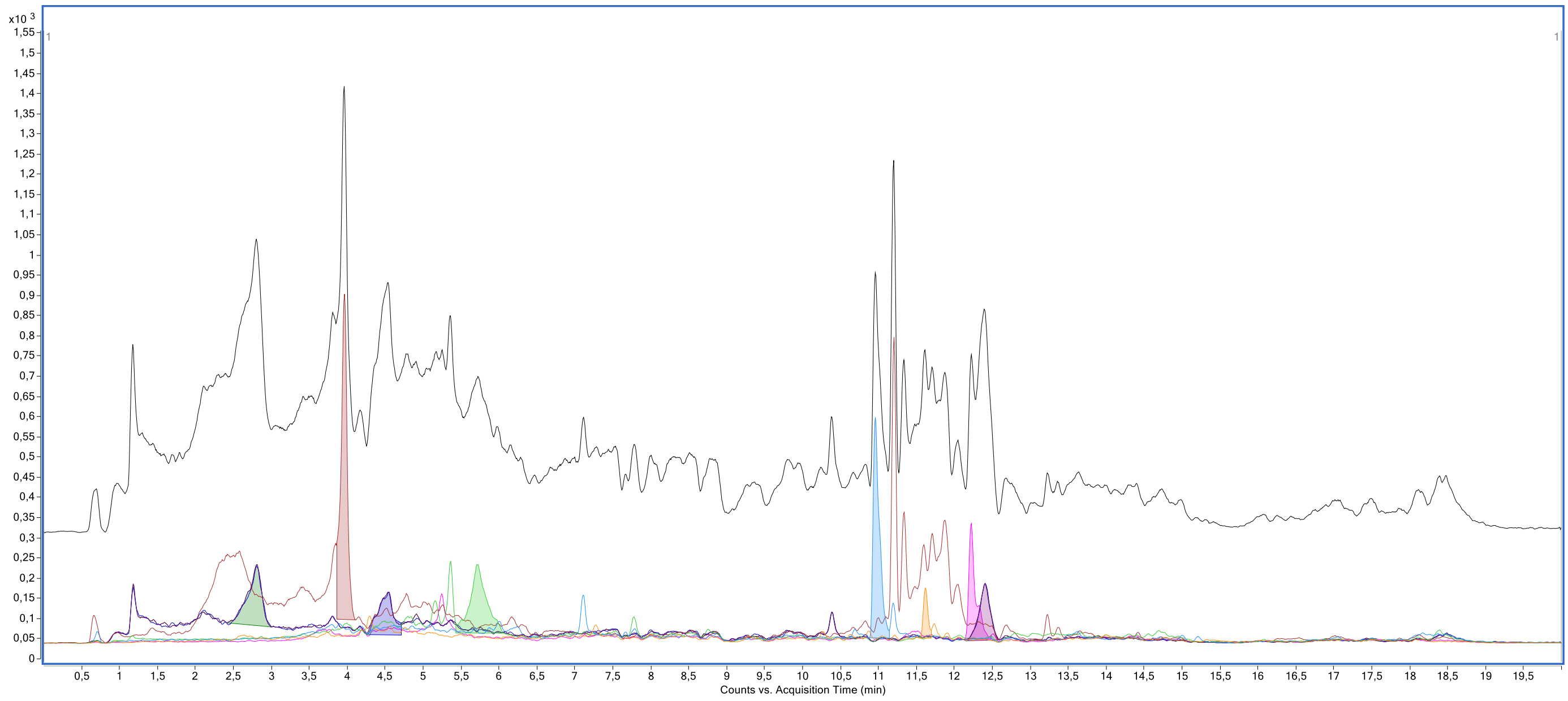

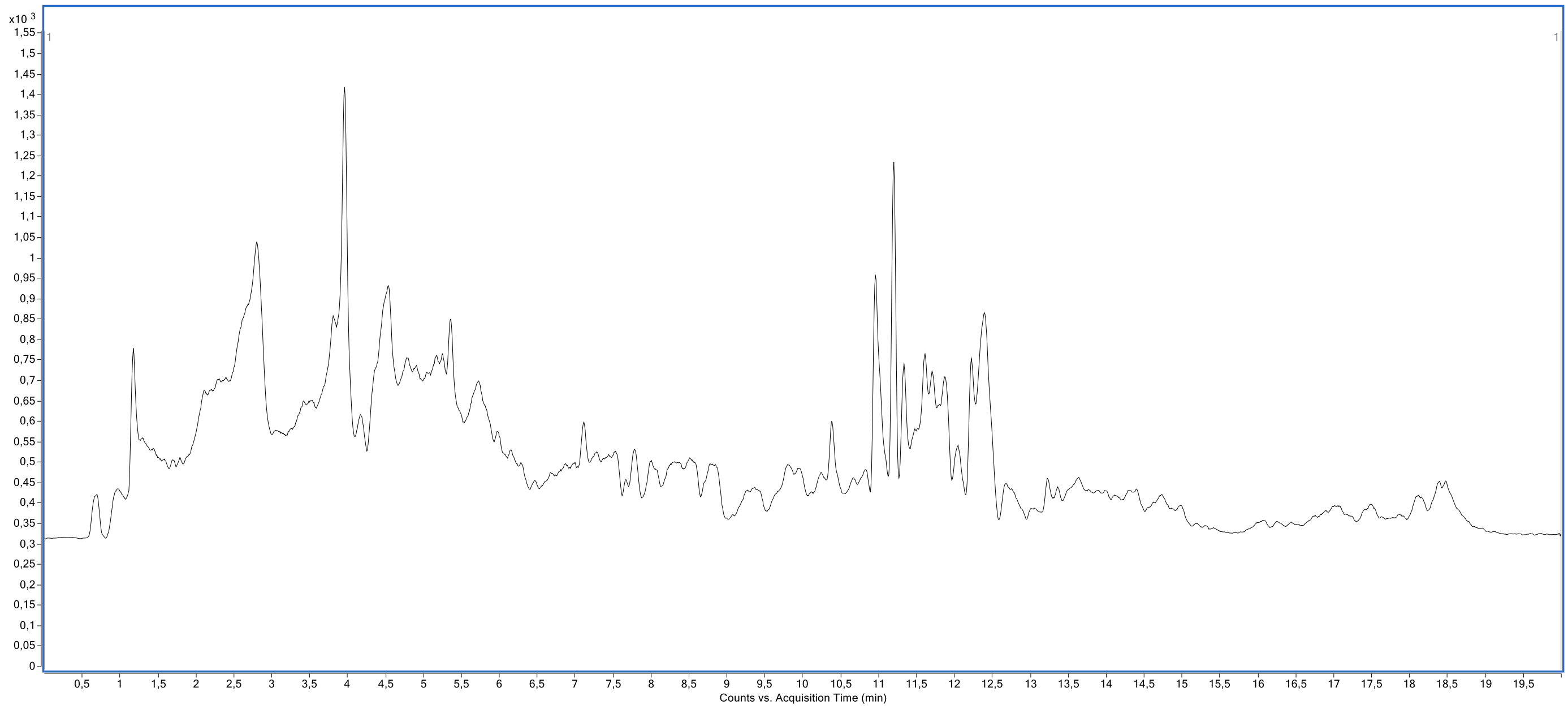

Total iyon kromatografisi (TIC)

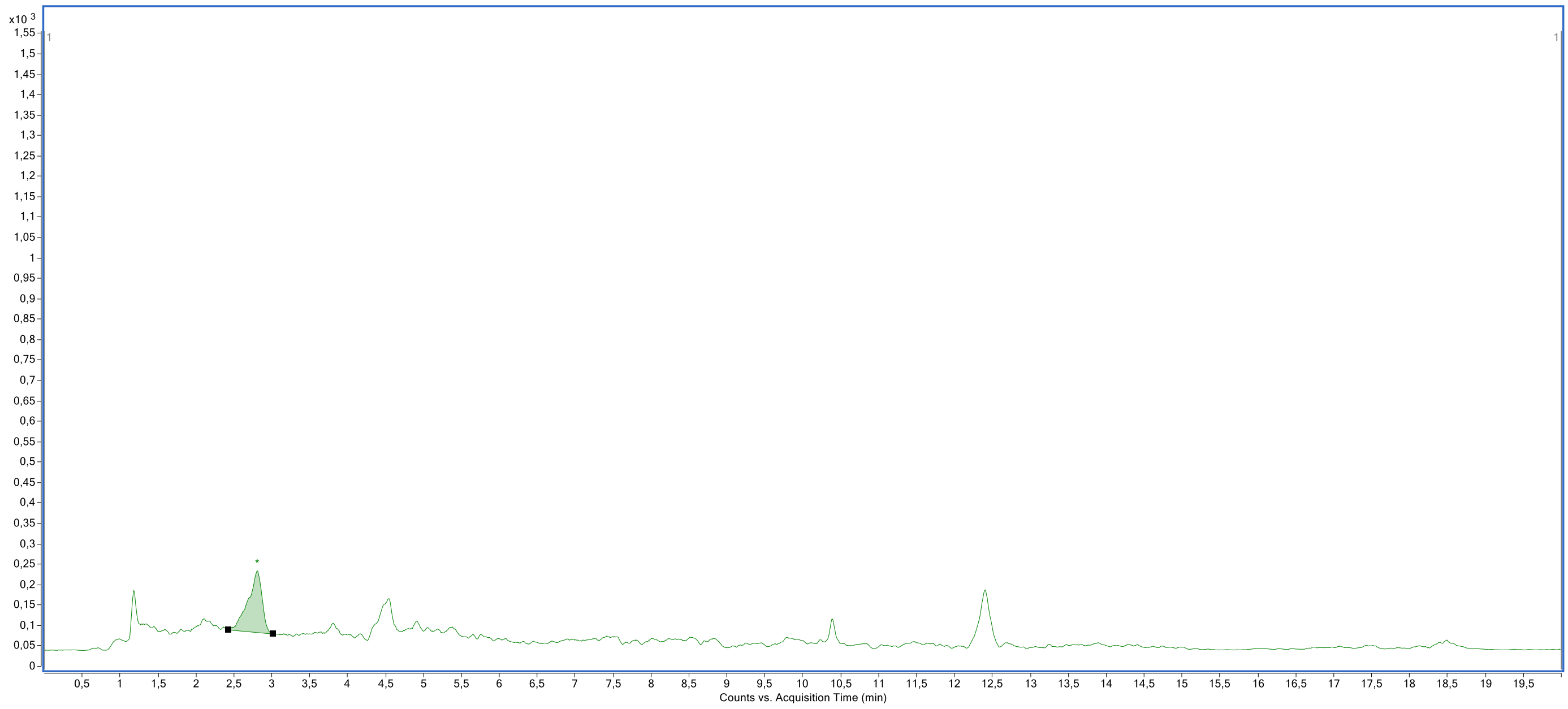

Beta-Carotene

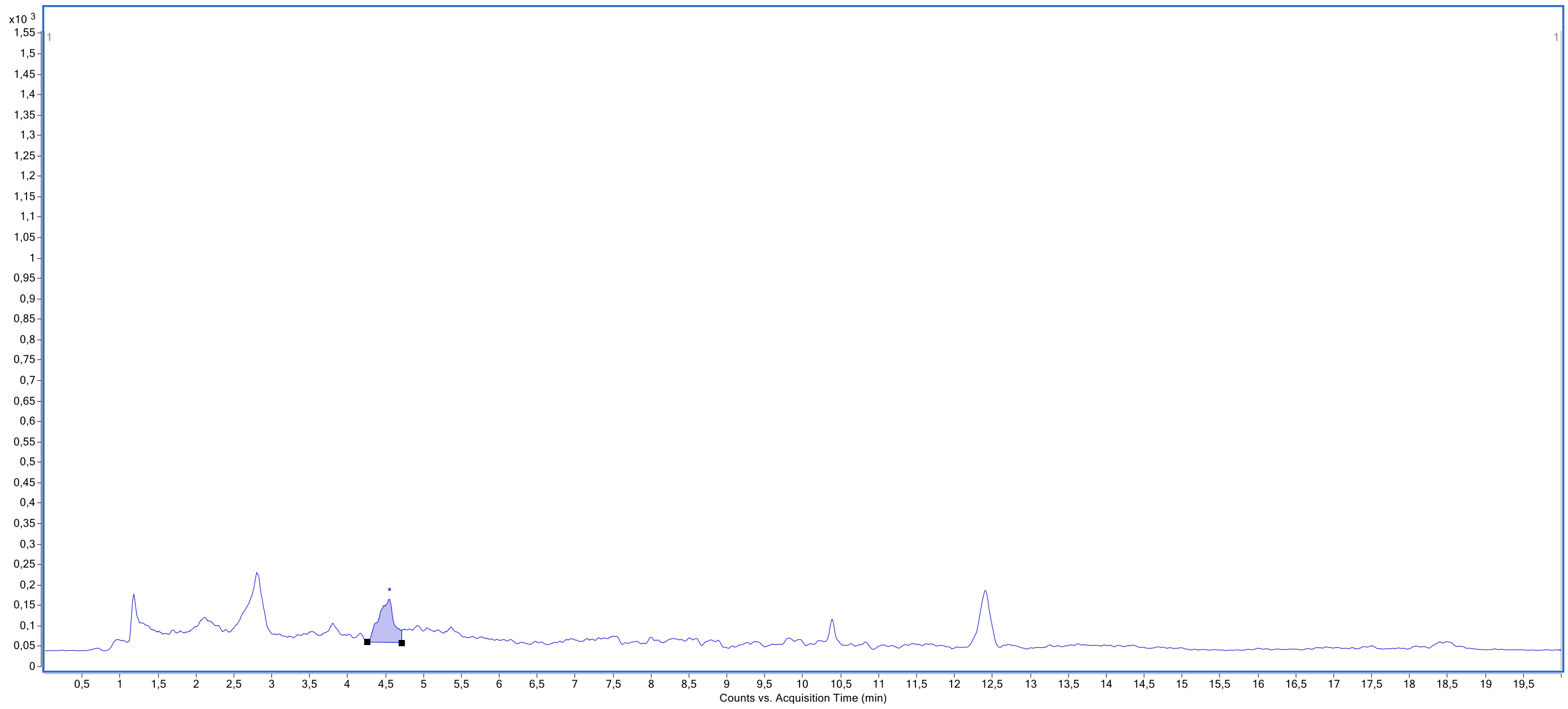

Alpha-Carotene

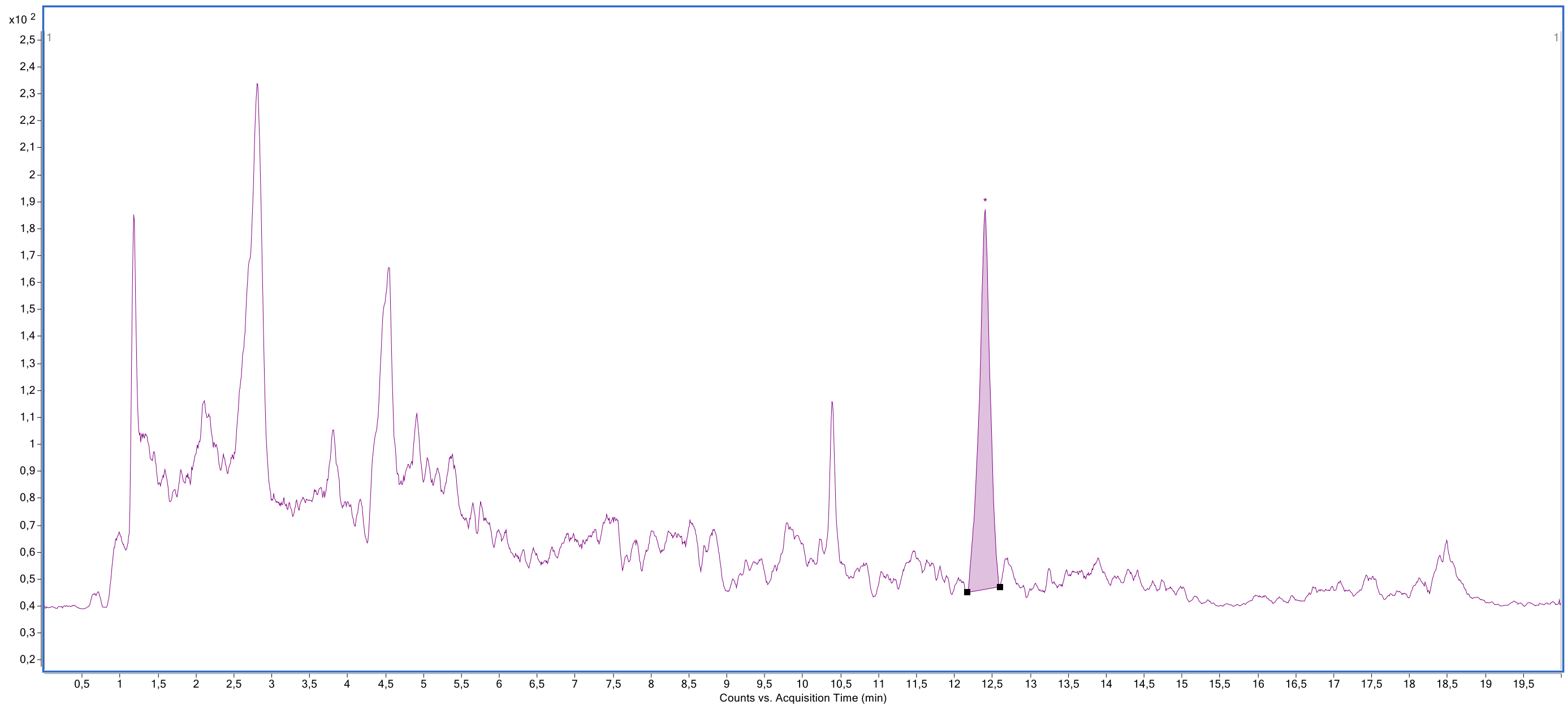

Lycopene

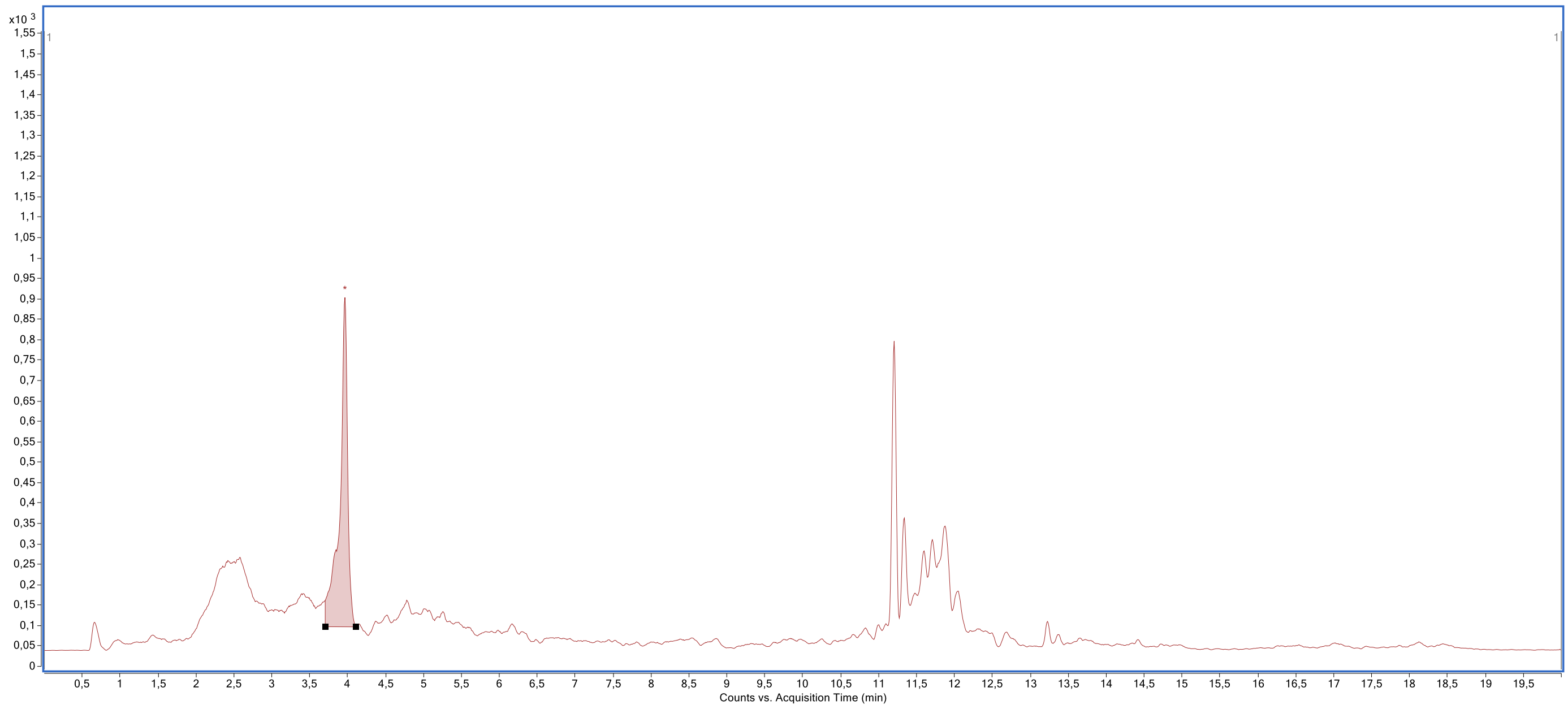

Phytoene

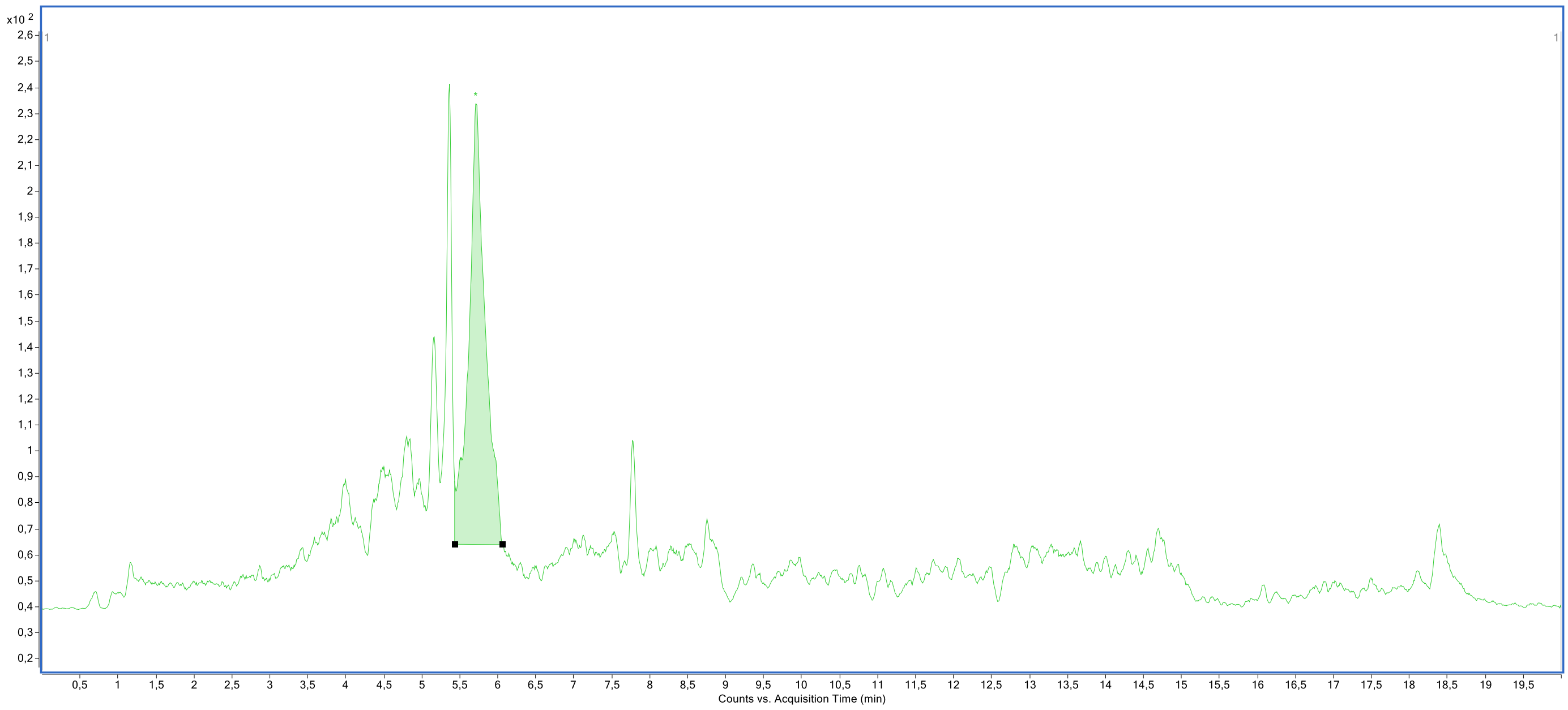

Beta-Cryptoxanthin

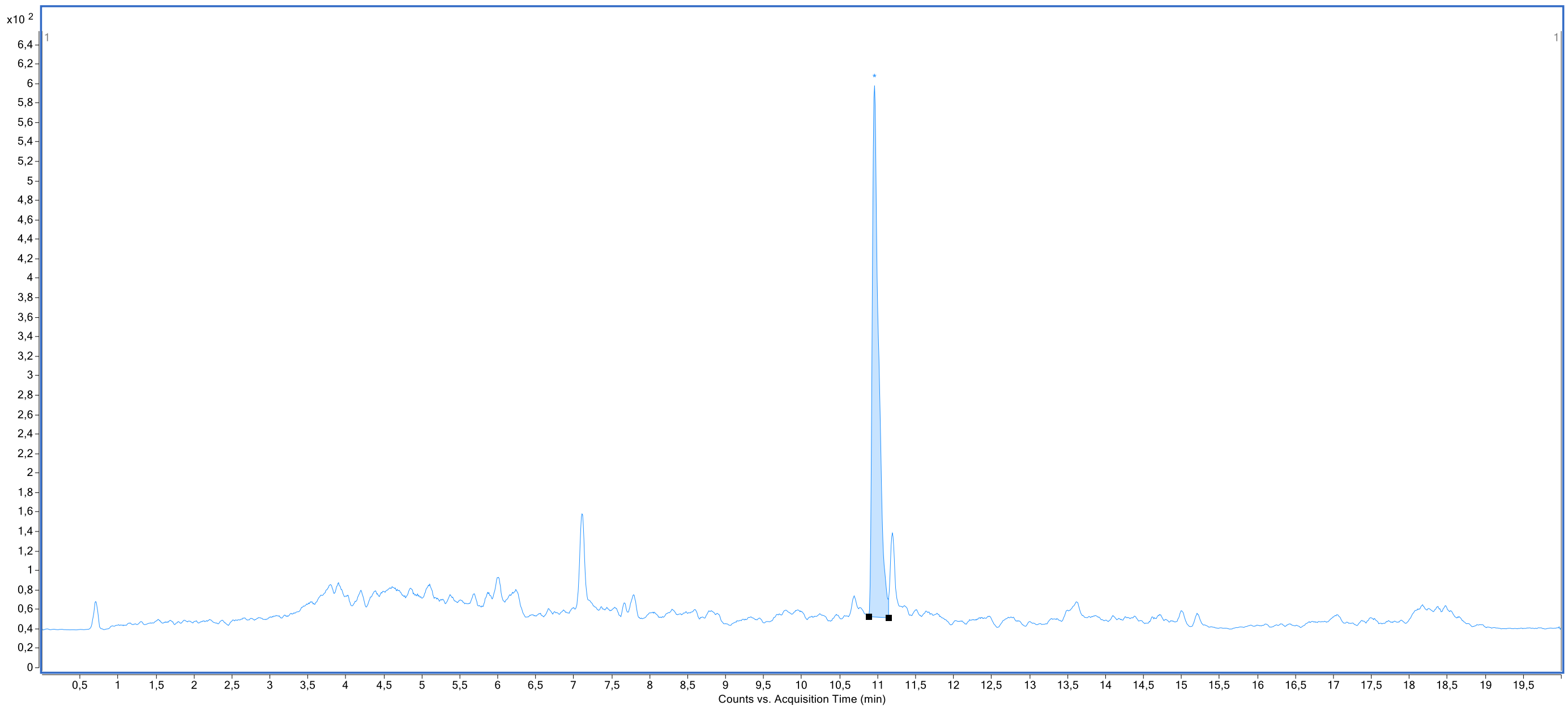

Zeaxanthin

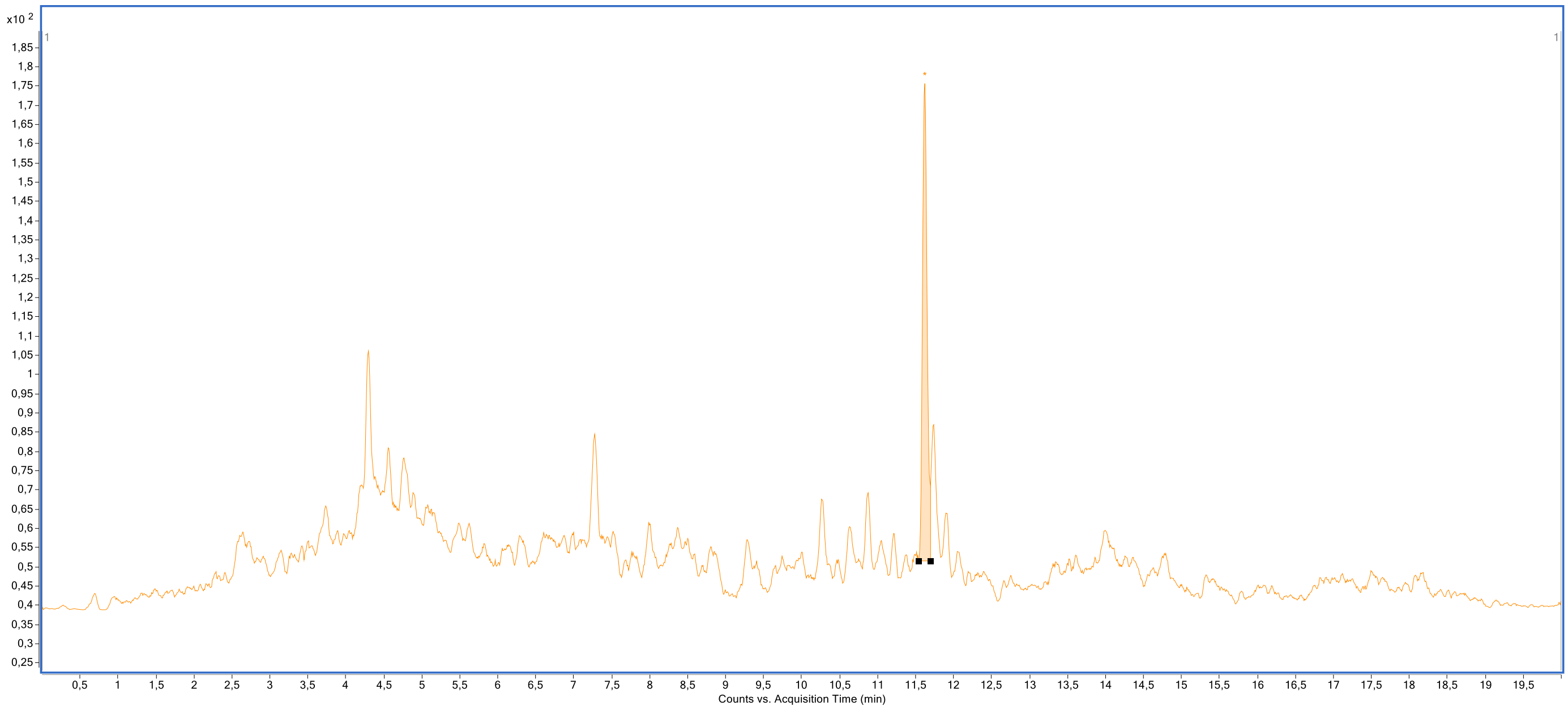

Sarcinaxanthin

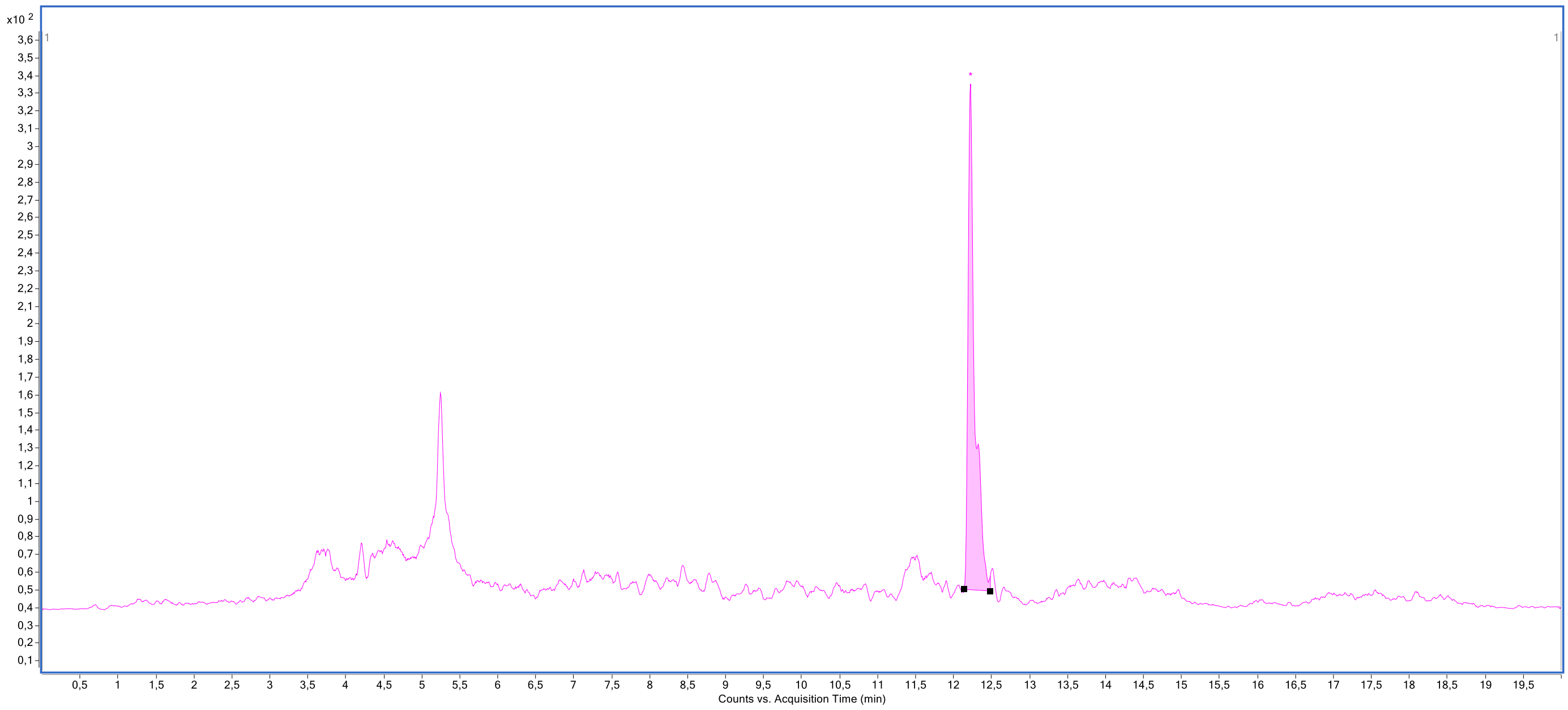

Astaxanthin

No	Product	Area	Area (%)
1	Beta-Carotene	1945	10.38
2	Alpha-Carotene	1501	8.01
3	Lycopene	1411	7.53
4	Phytoene	5803	30.97
5	Beta-Cryptoxanthin	2567	13.70
6	Zeaxanthin	3205	17.10
7	Sarcinaxanthin	534	2.85
8	Astaxanthin	1772	9.46
